# A Roadmap for Biomass‐Driven Development of Sustainable Phase Change Materials

**DOI:** 10.1002/cssc.202500288

**Published:** 2025-04-22

**Authors:** Alina Brzęczek‐Szafran, Magdalena Gwóźdź, Nicolas Brun, Marcin Wysokowski, Karolina Matuszek

**Affiliations:** ^1^ Faculty of Chemistry Department of Organic Chemical Technology and Petrochemistry Silesian University of Technology Krzywoustego 4 44‐100 Gliwice Poland; ^2^ ICGM, University of Montpellier, CNRS, ENSCM 34293 Montpellier France; ^3^ Institute of Chemical Technology and Engineering Faculty of Chemical Technology Poznan University of Technology Berdychowo 4 60965 Poznan Poland; ^4^ School of Chemistry Monash University Melbourne 3800 Australia

**Keywords:** biopolymers, carbon materials, composite, encapsulation, phase change material

## Abstract

While the world remains dependent on fossil fuels in nearly every aspect of life, unused biomass is piling up as waste, despite its significant potential for valuable applications—a critical missed opportunity for sustainable innovation. Phase change materials (PCMs) have emerged as a pivotal technology in the urgent transition toward carbon neutrality, especially considering that heating and cooling consume nearly half of global energy expenditure. This comprehensive review advances the scientific understanding of sustainability and circularity in PCM fabrication by providing a strategic framework for developing composites from renewable resources. This framework involves the introduction of a novel classification system (types 0–3) for biomass‐derived PCMs based on their levels of modification, enabling a comparison of material sources, performance metrics, and environmental impacts. By showing recent innovative developments in PCM shape stabilization, thermal conductivity enhancement, and leakage protection, it critically highlights the opportunities to replace conventional materials with innovative biomass‐derived alternatives, such as biomass‐derived carbons and polymers. Furthermore, the study integrates tools aligned with the Principles of Green Chemistry to aid the fabrication of truly sustainable materials, helping to guide researchers through material selection, process optimization, and the comprehensive evaluation of the environmental impact associated with their use and disposal.

## Graphical abstract

This comprehensive review advances the scientific understanding of sustainability and circularity in phase change material (PCM) fabrication by providing a strategic framework for developing composites from abundant, renewable resources. By screening biomass‐derived PCMs, carbons, and biopolymers, a roadmap is outlined for the development of sustainable composites with enhanced thermal conductivity and reduced leakage for thermal energy storage.

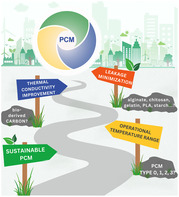

## 1 Introduction

The urgent need for sustainable materials and processes has become paramount as humanity faces unprecedented environmental challenges, including climate change, resource depletion, and waste accumulation. The continued reliance on fossil fuel‐based materials has led to cascading environmental impacts, from greenhouse gas emissions to microplastic pollution in our oceans and soil.^[^ ^1^ ^]^ This sustainability imperative has fundamentally transformed materials science research, driving innovation toward biobased alternatives that can and should replace petroleum‐derived products.^[^ ^2^ ^]^ Beyond its environmental benefits, this transformation is also driven by economic factors, as volatile fossil fuel prices and supply chain disruptions highlight the risks of petroleum dependency. Thermal energy storage (TES) materials are at the forefront of this transformation, playing a crucial role in renewable energy systems, energy‐efficient buildings, and sustainable industrial processes. These materials are particularly important as they address one of the key challenges in the transition to renewable energy: the need to efficiently store and manage thermal energy to bridge the gap between energy availability and demand.^[^ ^3^, ^4^ ^]^

TES technologies can broadly collect heat from any source and store it through three distinct mechanisms, which leverage different physical and chemical properties. The simplest mechanism is sensible heat storage (SHS), which utilizes the heat capacity of a material to collect heat as its temperature increases. It requires a wide range of temperatures to achieve reasonable storage capacity, but it is inexpensive, easily available, and already implemented in industry. SHS examples include alloys, rocks, molten salts, and thermal oils.^[^ ^5^ ^]^

Latent heat storage (LHS) operates under almost isothermal conditions, offering significantly higher energy density by utilizing phase change materials (PCMs) that absorb or release energy during phase transition (solid–solid, solid–liquid, or liquid–gas). Common PCMs include paraffin waxes,^[^ ^6^ ^]^ fatty acids,^[^ ^7^ ^]^ salt hydrates,^[^ ^8^ ^]^ polymers,^[^ ^9^ ^]^ metal alloys,^[^ ^10^ ^]^ sugar alcohols,^[^ ^11^ ^]^ and organic salts,^[^ ^12^, ^13^ ^]^ each offering different melting points and thermal properties to suit various applications.^[^ ^12^, ^14^ ^]^ However, each of these materials suffers specific drawbacks that hinder its practical applications: flammability, low stability, low thermal conductivity, phase separation, supercooling, or high price are among the most common.

The third mechanism, thermochemical heat storage (THS), leverages reversible chemical reactions to store and release thermal energy or to generate electricity.^[^ ^5^ ^]^ This method offers the highest theoretical energy density among all thermal storage approaches and can store heat for extended periods with minimal heat losses. THS includes chemical reaction storage utilizing: 1) hydration/rehydration reactions (e.g., CaO/H_2_O; MgO/H_2_O), 2) redox reactions (e.g., BaO/O_2_; CoO/O_2_), and 3) carbonization (e.g., CaO/CO_2_; MgO/CO_2_). Secondly, THS storage involves chemical adsorption (chemisorption) between metal halides and NH_3_ (e.g., CaCl_2_/NH_3_) or salt hydrates and H_2_O (e.g., MgCl_2_/H_2_O). Thirdly, THS encompasses chemical absorption (liquid‐gas absorption), including two‐phase absorption (e.g., H_2_O/NH_3_) or three‐phase absorption (e.g., LiCl solution + crystal/H_2_O).^[^ ^15^ ^]^

Recently, researchers have recognized that integrating all three thermal modes (sensible, latent, and thermochemical) into a single system can achieve exceptionally high TES capacities. At first, researchers combined two materials into a coworking matrix, e.g., a high‐density polyethylene (HDPE) as a PCM, and MgSO_4_·7H_2_O as a thermochemical material, with this system operating from 30 to ≈150 °C.^[^ ^16^ ^]^ A recent breakthrough revealed that the eutectic mixture of boric acid and succinic acid can undergo simultaneous melting and dehydration, making it the first known material to exhibit trimodal TES behavior with an exceptionally high energy storage capacity of almost 400 J g^−1^.^[^ ^17^ ^]^

Despite significant progress in materials and relevant TES technologies, the vast majority of these materials are derived from fossil fuels. Replacing fossil fuel‐based chemicals, materials, and products with biomass‐derived alternatives is one of the strategies that can help to achieve net zero emission targets. Current efforts to achieve this goal have extended beyond academic institutions and scientific research, encompassing state agencies, future policy development, and the regulation of rights associated with the acquisition, processing, and utilization of biomass. An example is the Biomass Strategy adopted by the UK, the first country whose government has undertaken a policy aimed at utilizing sustainable biomass across multiple sectors of its economy.^[^ ^18^ ^]^ Globally, sustainable consumption and production is prioritized through the Sustainable Development Goals (SDGs) proposed by the United Nations,^[^ ^19^ ^]^ which serve as a framework guiding the decision‐making processes for numerous governments.

The growing demand for biomass‐derived products is driving the development of advanced technological solutions for breaking down biomass (including biomass waste) into value‐added chemicals. Biorefineries play an important role in the production and delivery of such components. With the increasing number of operational facilities, valuable biomass‐derived products will become more readily available for the development of sustainable materials and products, including those for TES.

**Figure 1:**
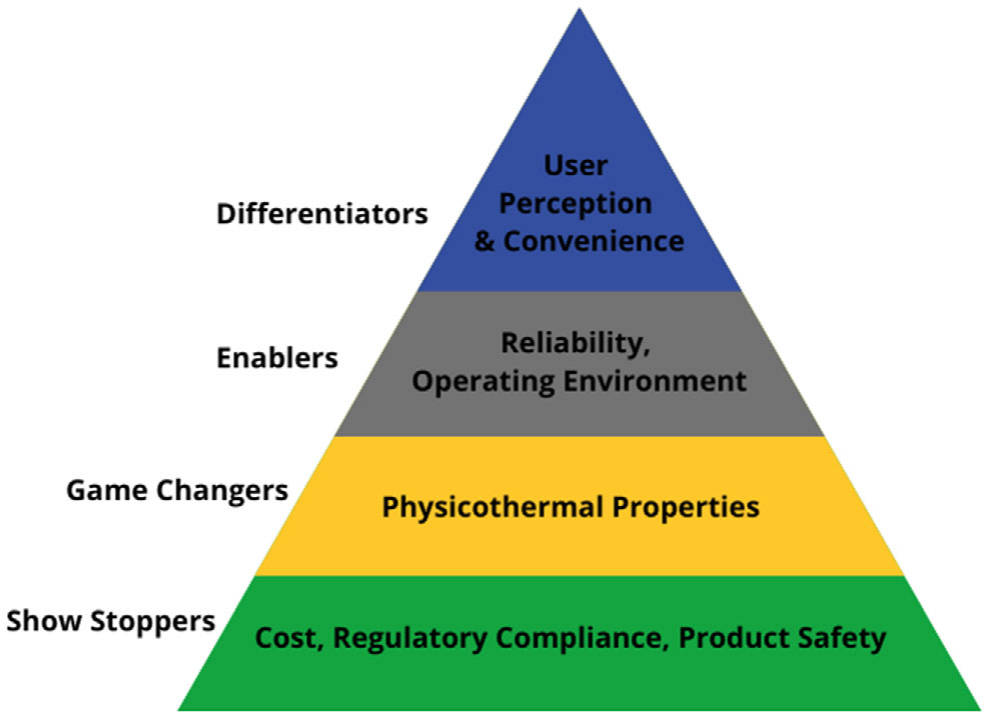
General selection characteristics of PCMs for TES applications proposed by Nazir et al. Adopted with permission.^[^ ^20^ ^]^ Copyright 2019, Elsevier.

With this review article, we aim to focus on PCMs that hold significant potential in a variety of applications, from cold chain storage to large‐scale renewable energy storage, while also offering strong prospects of being sourced from renewable biomass. Choosing the ideal PCM for TES requires carefully balancing various factors, such as operating temperature, high energy storage capacity, thermal and chemical stability, cost‐effectiveness, product durability, and overall sustainability. Nazir et al. organized this selection process by proposing a pyramid model for PCMs (**Figure** **1**).^[^ ^20^ ^]^ The pyramid's foundation addresses cost, regulatory compliance, and toxicity barriers. The middle section emphasizes thermal and physical properties that influence performance, while also accounting for reliability and environmental factors, such as cycling stability and material degradation. At the top are secondary considerations, such as user experience and convenience.

Even though PCMs have been investigated for nearly 50 years, a strong emphasis on their overall sustainability, including their production and utilization, has only become widespread recently. Many PCMs with a non‐renewable character, such as paraffin waxes, have the potential to be substituted with ones from renewable resources, such as vegetable oils, animal fats, or natural waxes.^[^ ^21^, ^22^ ^]^ Moreover, organic PCMs often suffer from insufficient thermal conductivity or inherent leakage problems, which has directed research toward the construction of composites. Combining PCMs with thermally conductive fillers, such as carbon materials (carbon nanotubes,^[^ ^23^ ^]^ graphene,^[^ ^24^, ^25^ ^]^ graphene oxide,^[^ ^26^ ^]^ graphite,^[^ ^26^ ^]^ carbon nitride^[^ ^27^ ^]^) or boron nitride,^[^ ^28^ ^]^ has emerged as a solution to enhance organic materials’ thermal conductivity and prevent leakage by the development of shape‐stabilized materials. Moreover, encapsulation of the PCM in a shell made of polymer, silica, alumina, metals, or hybrid material is a strategy that can minimize the leakage problem. Materials addressing both of these issues have a strong potential to be sourced from biomass, giving rise to fully biobased composites, paving the way toward new combinations in the field of material science.

Beyond developing PCMs from biomass‐derived materials, researchers are also creating biomimetic PCMs that mimic natural biological systems.^[^ ^29^ ^]^ These include structures mimicking those known from nature that can further provide additional functionality to PCMs (e.g., honeycomb structures with excellent mechanical properties and high porosity or structures inspired from spider webs providing fibers with strong adhesion and tensile abilities) as well as working themselves in terms of thermal management. For more insights into nature‐inspired strategies, we recommend referring to the excellent review article by Liu et al.^[^ ^30^ ^]^

With the increasing awareness of the development of sustainable materials,^[^ ^31^ ^]^ we aim to highlight the idea of biomass‐derived PCM composite fabrication as an overall concept using biomass‐derived precursors at all stages. This review examines widely available bioderived materials for TES applications, including biomass‐derived PCMs, biomass‐derived carbons for thermal conductivity enhancement, and natural polymers suitable for the encapsulation process to create fully bioderived, efficient composite materials that operate across various temperature ranges. Finally, we will highlight the most effective tools for assessing the overall sustainability of materials and supporting decision‐making in selecting specific materials and unit operations during the fabrication process.

## 2 Biomass‐Derived PCMs

Organic PCMs encompass a diverse range of compounds, including paraffin waxes, alkanes, fatty alcohols, acids, esters, ureas, amides, urethanes, carbonates, ionic liquids/organic salts, and sugars and their derivatives. Some of these materials have a strong potential to be fully or partially developed using renewable resources. The costs associated with biomass processing and upgrading can still be less competitive compared to petroleum‐based alternatives, taking into consideration, among other factors, the undeveloped feedstock delivery chains, the precursors’ seasonal and geographical availability, and the necessary investment in infrastructure and technologies.^[^ ^21^, ^32^ ^]^ Nevertheless, the ongoing advances in sustainable biomass valorization methods, including separation and purification steps and raw material acquisition, as well as making use of waste (e.g., waste cooking oil), make biomass‐derived chemicals, including bio‐PCMs, more affordable and practical, fostering a circular economy and promoting more sustainable TES applications.^[^ ^33^ ^]^

**Figure 2:**
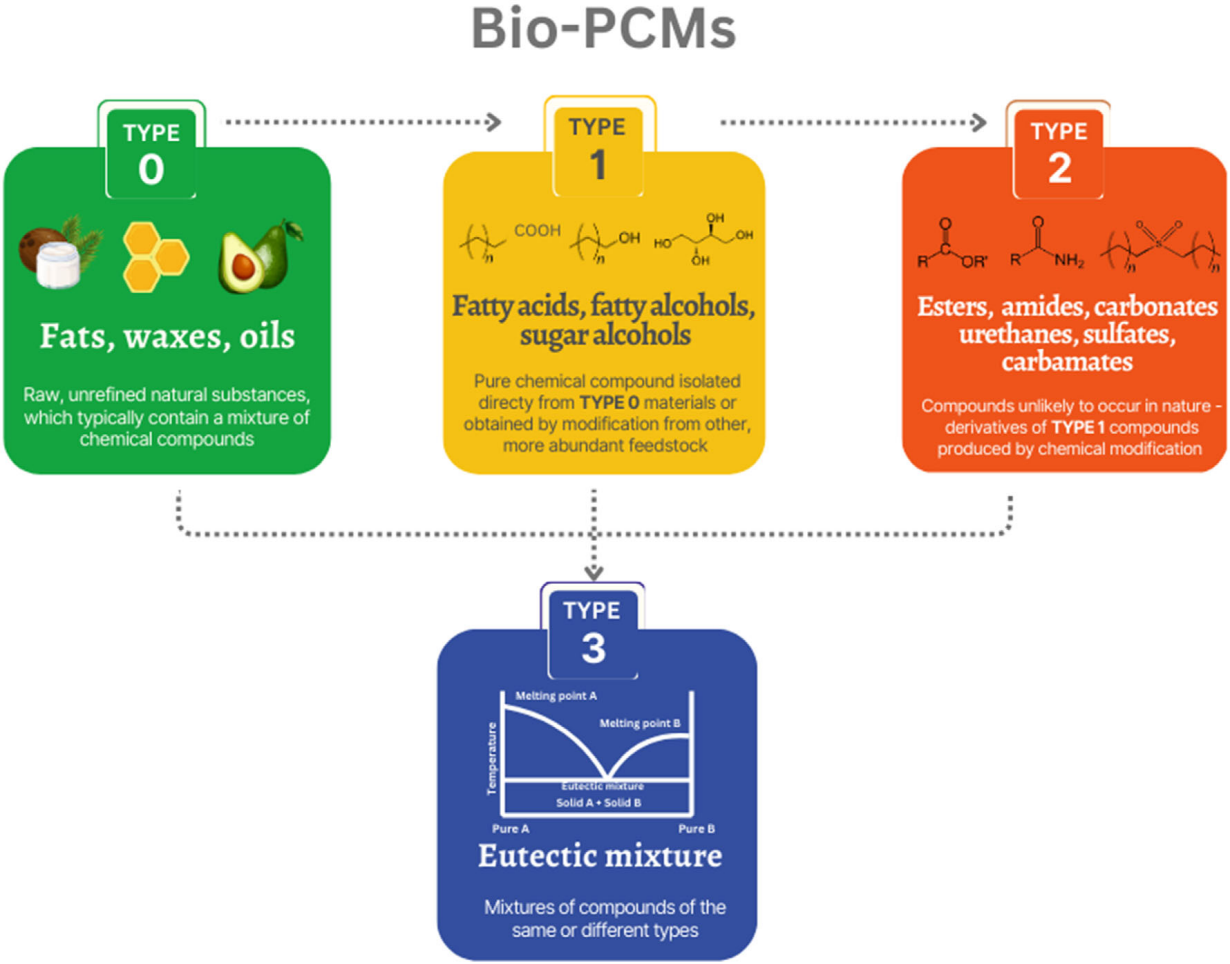
Biomass‐derived PCMs categorized into four types, depending on their modification level.

To categorize and compare bio‐PCMs, based on their source, environmental impact, performance, and cost, we classified bio‐PCMs into four types: 0, 1, 2, and 3, depending on their modification level (**Figure** **2**). Type 0 bio‐PCMs are raw, unmodified mixtures of various compounds mainly extracted from vegetable oils, waxes, or fats. Type 1 bio‐PCMs are single compounds isolated from these mixtures, including fatty acids, fatty alcohols, and sugar alcohols. Their concentration in nature is usually low, so they are mostly obtained by modification of other, more abundant feedstocks (triglycerides or lignocellulosic biomass). Type 2 bio‐PCMs include compounds that are unlikely to occur in nature. These are derivatives (esters, amides, urethanes, carbonates, sulfates, or carbamates) of type 1 bio‐PCMs. The degree of chemical modification of this group is the highest. Type 3 bio‐PCMs are eutectic mixtures of compounds of different types. The increasing number of unit operations performed for each successive material type is accompanied by introducing improvements to overcome the limitations of the precursor. As we provide a state‐of‐the art review, we focus only on materials already recognized as applicable to TES in literature.

### 2.1 Type 0 Bio‐PCMs

Type 0 PCMs (**Table** **1**) consist of raw, unrefined natural substances that typically contain a mixture of compounds, such as fatty acid triglycerides, sterols, waxes, fatty acids, fatty acid esters, alcohols, ketones, aldehydes, and other trace components.^[^ ^34^, ^35^ ^]^ To obtain these compounds, only the extraction (cold pressing, solvent extraction, or mechanical expelling) of vegetable oil from its biological source is usually required, preceded by several operations (hulling, crushing, pressure‐filtration, etc.) at very limited cost.^[^ ^21^ ^]^ In some cases, such as beeswax, no additional operations are required. It is essential to select the extraction technique that minimizes environmental impact while maximizing yield.

**Table 1:** Thermal properties of TYPE 0 bio‐PCMs.

Type 0 bio‐PCMs					
Natural oils, waxes, or fats		*T*_m_ [°C]	Δ*H*_f_ [J g^−1^]	Isolation process	References
Shellac wax		51–82	148	Extracted from shellac produced by *Kerria lacca* insects	[237]
Beeswax		38–68	206	Produced in beehives by honeybees	[39, 238]
*Calophyllum inophyllum* seed oil		−6–14	184	Extracted from the tamanu tree's kernel	[239]
Avocado oil		−20–12	70	Extracted from avocado	[21]
Argan oil		−30–7	52	Extracted from fruit of the argan tree
Coconut oil		6–32	133	Extracted of coconut kernel
Jojoba oil		−5–18	142	Extracted from jojoba kernel
Soybean oil		−33–(−12)	50	Extracted from soybean
Triglycerides	Glycerol tripalmitate	59	186	Extracted from plants oils, fats, waxes	[240]
	Glycerol tribehenate	82	214		[241]

Vegetable and animal oils and fats remain key renewable resources for the chemical industry. Over the last decade, their industrial use increased from 31 to 51 million metric tons annually.^[^ ^36^ ^]^ These abundant raw materials serve as a crucial resource for the sustainable production of biobased PCMs, supporting broader industrial and environmental goals.

The composition and resulting thermal properties of materials derived from oleochemical feedstocks, such as melting and crystallization temperatures and related enthalpies, depend on external factors, such as environmental conditions and extraction methods.^[^ ^37^ ^]^ Due to their multicomponent nature, type 0 bio‐PCMs often exhibit wider melting ranges,^[^ ^38^ ^]^ with multiple melting and crystallization peaks, making them suitable for applications where a precise melting range is not essential. Their inherent nontoxicity and reduced flammability make them safe for use in building materials, solar air heaters, and solar water heaters.^[^ ^21^ ^]^

Recently, Hammami et al.^[^ ^21^ ^]^ characterized the thermal properties of 45 vegetable oils as PCMs. Most of the oils gave Δ*H*_f_ values <100 J g^−1^ and wide melting ranges (<50 °C), but there were exceptions that gave higher enthalpies of fusion, such as jojoba oil, palm kernel oil, and coconut oil with Δ*H*_f_ values of 142, 140, and 133 J g^−1^ respectively. Dinker et al.^[^ ^39^ ^]^ studied beeswax with a high enthalpy of fusion reaching 206 J g^−1^, measured over a broad temperature range (from 38 to 61 °C), for low‐temperature thermal storage as an alternative to synthetic paraffins. While natural oils and waxes are generally thermally stable, their variable compositions can limit their consistency and reliability in specific applications. Furthermore, these materials often have lower thermal conductivity, undergo volume changes during phase transitions, are flammable, and may be expensive or difficult to obtain, as is the case with certain seed oils. Animal fats, on the other hand, are highly susceptible to oxidation and hydrolysis, which leads to rapid degradation and reduced thermal efficiency over time, thus limiting their shelf life.^[^ ^37^ ^]^

Oils and waxes are mainly composed of triglycerides, and some, such as cocoa butter, consist entirely of them. Hence, we classified triglycerides as type 0 bio‐PCMs. Ravotti et al.^[^ ^40^ ^]^ recently highlighted the potential of triglycerides as effective candidates for TES applications by characterizing their thermal properties individually. These esters offer a diverse melting temperature range from below 0 °C to above 90 °C and in some cases exhibit Δ*H*_f_ values exceeding 200 J g^−1^. However, a key challenge remains managing polymorphism (the ability of triglycerides to crystallize in different forms), which can affect their thermal stability and consistency. Overcoming this limitation is crucial to maximizing the practical use of triglycerides in TES systems. Further modification of natural oils through different approaches can improve their thermal properties and stability, further enabling their efficient operation in various applications.

### 2.2 Type 1 Bio‐PCMs

Biomass is a source of valuable compounds, including fatty alcohols, fatty acids, sugar alcohols, and lactones, that can serve as PCMs for TES applications. However, these compounds often appear in low concentrations in nature, so their extraction and purification can be costly. Type 1 bio‐PCMs are preferably derived from more abundant feedstocks (triglycerides and lignocellulosic biomass) through chemical transformations, including hydrolysis, transesterification, and hydrogenation, followed by additional purification steps, such as precipitation or crystallization (**Table** **2**). Fatty acids, for instance, are naturally present in vegetable oils and animal fats. Conventional chemical hydrolysis of triglycerides using acidic or basic catalysts can be utilized or hydrothermal, enzymatic, or biochemical processes that offer improvements by avoiding large amounts of contaminated waste and vessels made from expensive corrosion‐resistant materials.^[^ ^41^ ^]^ Other valuable feedstocks for fatty acids are waste animal fat, non‐edible plant oils, and used cooking oil (UCO).^[^ ^32^ ^]^ For example, Kumar and Negi hydrolyzed UCO with a lipase from *Penicillium chrysogenum*, obtaining C_18_ fatty acids with 22% yield.^[^ ^42^ ^]^

**Table 2:** Thermal properties of TYPE 1 bio‐PCMs.

TYPE 1 bio‐PCMs						
Group of compounds	Compound	*T*_m_ [°C]	Δ*H*_f_ [J g^−^1]	Origin	Production processes	References
Fatty acids	Capric acid	32	169	Triglyceride	Extraction from plant natural oils Hydrolysis of triglycerides, with removal of glycerol	[43, 44, 242]
	Stearic acid	55	159			
Fatty alcohols	1‐Hexadecanol	49	237	Fatty acids	Hydrogenation of fatty acids Hydrogenation of methyl esters of fatty acids	[47]
	1‐Octadecanol	57	248			
Sugar alcohols	Erythritol	119	333	Starch, fruits such as melon, watermelon, pears, grapes; and in fermented foods, such as cheese, soy sauce	Fermentation of glucose	[49, 53]
	Mannitol	166	284	Starch, cellulose and hemicellulose, fructose, mushrooms, brown algae, tree bark	Catalytic/enzymatic hydrogenation of fructose Fermentation of fructose	
	Xylitol	92	233	Hardwoods, softwoods, agricultural waste from processing maize, wheat, rice	Catalytic/enzymatic hydrogenation of xylose Fermentation of xylose	

Fatty acids have been extensively studied due to their favorable thermal properties. They demonstrate melting points ranging from 32 °C for capric acid^[^ ^43^ ^]^ to 55 °C for stearic acid (90% purity),^[^ ^44^ ^]^ making them well‐suited for low‐temperature applications. Additionally, they have high enthalpies of fusion (some sources report enthalpy values for stearic acid as high as 258 J g^−1^^[^ ^45^ ^]^), further underscoring their thermal storage potential. Fatty acids offer durability, withstanding thousands of thermal cycles (melting and freezing) without significant degradation in performance.^[^ ^44^ ^]^ Other benefits include low supercooling, a narrow melting range, and stability. However, fatty acids face drawbacks, such as low thermal conductivity (0.2388 W m^−1^ K^−1^ for C12 and 0.3079 W m^−1^ K^−1^ for C18), an unpleasant odor after repeated cycles,^[^ ^20^ ^]^ potential metal corrosion,^[^ ^46^ ^]^ flammability, and a high sublimation rate. The phase change temperature and latent heat of PCMs can be influenced by a number of factors, so values of the thermal properties of fatty acids, as well as other bio‐PCMs reported in the literature, can vary slightly.

Fatty alcohols avoid some of the issues assigned to fatty acids, such as odor and corrosiveness. An example is the group of common fatty alcohols studied by Q. Zhang et al.^[^ ^47^ ^]^ which exhibited melting points varying from 23 °C for dodecanol to 57 °C for octadecanol, with enthalpies of fusion up to 248 J g^−1^.^[^ ^47^ ^]^ Depending on the oil feedstock, fatty alcohols with different chain lengths are produced. Nonetheless, their production is more complex than that of fatty acids, as it involves hydrogenation of fatty acids, fatty acid methyl esters (FAME), or fatty acid wax esters (FAWE).^[^ ^48^ ^]^ These transformations require metal catalysts and high energy consumption. An alternative is the biological route for producing fatty alcohols in the presence of enzymes.^[^ ^48^ ^]^ These processes are more eco‐friendly, being metal‐free, energy‐efficient, and not requiring high pressure, but still require optimization for industrial‐scale applications.

Sugar alcohols, with melting points reaching 190 °C and enthalpies up to 350 J g^−1^ for galactitol, represent a unique subset of medium‐temperature PCMs, especially suitable for renewable energy storage.^[^ ^49^ ^]^ They are commonly present in a variety of fruits and vegetables, while their industrial production involves the catalytic hydrogenation of sugars. The recent drive for advances in chemical transformations that enable milder reaction conditions,^[^ ^50^ ^]^ alongside biotechnological approaches such as microbial fermentation, has contributed to the sustainable development of sugar alcohols.^[^ ^51^ ^]^ Despite favorable thermal properties, including the highest enthalpies of fusion among organic compounds that melt in the intermediate temperature range, sugar alcohols encounter challenges, such as severe supercooling and crystallization issues, which hinder complete heat retrieval and their operational stability. These materials undergo 10%–15% expansion upon melting,^[^ ^52^ ^]^ while their solid‐state thermal conductivities range from 0.37 to 1.31 W (m·K)^−1^ for xylitol, depending on the measurement technique.^[^ ^53^, ^54^ ^]^ Improvements to overcome crystallization challenges and enhance the stability of sugar alcohols over repetitive melting and crystallization are needed to fully utilize the potential of these renewable materials for broader PCM applications. However, their supercooling issue could be used as an advantage in applications such as seasonal energy storage.^[^ ^55^ ^]^

Carbohydrates, which possess hydroxyl‐group‐rich structures similar to sugar alcohols, along with significant energy storage density, also suffer from severe supercooling. Recently, Gaida et al. presented a novel strategy to reduce supercooling in polyol, carbohydrate‐derived PCMs by converting them into organic salts with anions that provide additional H‐bonding.^[^ ^56^ ^]^ Nevertheless, carbohydrates have been mostly studied only as PCM additives.^[^ ^57^, ^58^ ^]^

### 2.3 Type 2 Bio‐PCMs

While type 1 materials represent an improvement over raw type 0 bio‐PCMs, several challenges remain for the various groups of materials, including corrosion issues, supercooling, thermal stability, and operational temperature range. Some of these issues can be solved by further chemical modification of type 1 bio‐PCMs producing type 2 bio‐PCMs (**Table** **3**).

**Table 3:** Thermal properties of TYPE 2 bio‐PCMs.

Type 2 bio‐PCMs							
	Compound	*T*_m_ [°C]	Δ*H*_f_ [J g^−1^]	Origin		Production method	References
				Biomass	Fossil fuels^a^^)^		
Monoesters	Decyl eicosanoate	41	232	Fatty alcohol, fatty acid	–	Esterification	[65]
	Tetradecyl octadecanoate	49	222				[243]
	Octadecyl eicosanoate	65	226				[244]
	Threo‐dihydroxystearic esters	52–77	137–234				[37]
Diesters	Glycol distearate (Cutina AGS)	60	213	Fatty acid, ethylene glycol	–	Esterification	[245]
	Dihexadecyl‐1,10‐decanedioate	58	214	Sebacic acid, fatty alcohol	–	Esterification	[61]
	Dihexadecyl‐1,8‐ octanedioate	55	216	Octanedioic acid, fatty alcohol			[60]
	Dioctadecyl tartrate	82	221	Fatty alcohol, Tartaric acid	–		[75]
	Didocanosyl tartrate	94	203				
	Neopentyl glycol dipalmitate	35	118	Fatty acid	Neopentyl glycol		[62]
Polyol esters	Erythritol tetrapalmitate	22	201	Sugar alcohols, fatty acids	–		[246]
	Xylitol pentastearate	32	205				[72]
	Galactitol hexastearate	50	251				[73]
FAME	Methyl stearate	36	204	Plant oil, methanol	–	Transesterification	[64]
Lactones	ε‐Caprolactone	−1.6	122	6‐Hydroxyhexanoic acid	Cyclohexanone	Lactonization/Baeyer–Villiger oxidation	[87]
	γ‐Valerolactone	−32	110	Levulinic acid	–	Catalytic hydrogenation and dehydration	
Carbonate	Tetradecyl carbonate	34	227	Fatty alcohol, diethyl carbonate	–	Carbonate interchange reaction	[77]
	Octadecyl carbonate	52	223				
Sulfones	1‐(Decyl sulfonyl) decane	89	204	Fatty sulfides	–	Oxidation	[76]
	1‐(Octadecyl sulfonyl) octadecane	109	218				
Fatty amineb)	1‐Dodecylamine	27	261	Fatty nitriles	–	Hydrogenation	[79]
	1‐Hexadecylamine	45	280				
Monoamide	Fatty acid amides from sunflower oil and dodecylamine	49	140	Sunflower oil, fatty acid	–	Condensation	[80]
	*N*‐hexadecyl‐decanamide	79	201	Fatty acids	Amine		[79]
Diamide	*N, N′*‐(ethane‐1,2‐diyl) dipalmitamide	151	152				[247]
	*N, N′*‐(octane‐1,8‐diyl) distearamide	144	218				[81]
Dicarbamate	Dioctadecyl hexane‐1,6‐dicarbamate	121	200	Fatty alcohol	Diisocyanate	Alcoholysis	[82]
	Dioctadecyl phenyl‐1,6‐dicarbamate	151	135				
Organic salts	Choline tartrate	151	129	Tartaric acid, choline chloride^c^^)^	–	Anion metathesis, acid‐base reaction	[248]
	2‐hydroxyethylammonium stearate	76	176	Fatty acid	Amine	acid‐base reaction	[249]

a) A compound derived from fossil fuels or produced through reaction with a fossil fuel–based compound.

b) Although amines are precursors of amides and could be classified as type 1, they do not occur naturally and were ultimately classified as type 2 as typically synthetic compounds.

c) Despite that choline chloride can theoretically be extracted directly from a biomass, it is industrially produced from fossil fuels.

Type 2 bio‐PCMs include esters, amides, urethanes, carbonates, sulfates, carbamates, and organic salts/ionic liquids. Unlike their precursors, these derivatives are rarely present in nature and are primarily produced through synthetic processes. Their production involves additional processing steps and unit operations, which increase both costs and environmental impact. However, these derivatives offer PCMs with much broader melting temperatures.

An important group of type 2 bio‐PCMs are aliphatic esters, particularly those derived from fatty acids, fatty alcohols, diols, or diacids, which have been extensively studied by Aydin et al.^[^ ^59^, ^60^, ^61^ ^]^ Sari,^[^ ^62^, ^63^ ^]^ and Ravotti.^[^ ^64^, ^65^ ^]^ These esters are useful as low‐temperature PCMs with low corrosivity, little unpleasant odor, and improved thermal stability compared to fatty acids. Common synthetic methods include Fischer esterification and transesterification, which can both be operated at low cost, offering high substrate flexibility.^[^ ^66^, ^67^ ^]^ While there are numerous reviews detailing aliphatic esters,^[^ ^68^ ^]^ certain examples stand out for their exceptional properties, such as decyl eicosanoate, which boasts an enthalpy of 232 J g^−1^.^[^ ^65^ ^]^ However, polymorphism remains an issue, albeit less pronounced than in triglyceride‐based PCMs.^[^ ^69^ ^]^

Another group of facile bio‐PCMs for low‐temperature applications are FAMEs. These compounds are typically produced through oilseed extraction, followed by a subsequent alkali‐catalyzed reaction between the plant oils and methanol.^[^ ^70^ ^]^ An example is methyl stearate with a melting point of 36 °C and Δ*H*_f_ value of 204 J g^−1^, reported by Ravotti et al.^[^ ^64^ ^]^

Esterification of sugar alcohols with fatty acids to give the corresponding esters (erythritol tetrapalmitate,^[^ ^71^ ^]^ xylitol pentastearate,^[^ ^72^ ^]^ or galactitol hexastearate^[^ ^73^ ^]^) addresses the challenges of supercooling and low stability, which are often observed for sugar alcohols. The highest enthalpy, reaching 250 J g^−1^, along with a melting point of 50 °C, was reported for galactitol hexastearate, showing a decrease in stability (≈17%) over 1000 heating and cooling cycles.^[^ ^73^ ^]^ Despite an improvement in cycling stability, this modification offers materials with melting temperatures similar to that of fatty acids rather than sugar alcohols, limiting their practical use to low‐temperature applications.

In addition to addressing the limitations of the precursor materials, many efforts have focused recently on studying the effect of different functional groups that impact the melting range, thereby enhancing the potential applications of PCMs.^[^ ^74^ ^]^ For instance, we have demonstrated that by introducing hydroxyl groups into the fatty esters by using renewable tartaric acid as the core, it is possible to increase the melting temperature relative to the compound without —OH groups. Ester‐derived behenyl alcohol melts at 94 °C with an enthalpy of 203 J g^−1^, demonstrating excellent stability over 500 heating and cooling cycles.^[^ ^75^ ^]^

Another class of promising PCMs for medium‐temperature TES applications are sulfones, which typically have slightly higher phase transition temperatures, as reported for octadecyl sulfone (*T*_m_ = 109 °C, Δ*H*_f_ = 200 J g^−1^).^[^ ^76^ ^]^ These compounds can be produced by simple solvent‐free oxidation of the fatty sulfides using hydrogen peroxide and acetic acid, but the challenge remains the cost of the fatty sulfides.

On the other hand, carbonates with melting points ranging from −2 °C for decyl carbonate to 53 °C for octadecyl carbonate are dedicated for low‐temperature TES.^[^ ^77^ ^]^ These values are significantly lower than those of fatty acids, fatty alcohols, n‐alkanes, and wax esters with similar carbon chain lengths. Most carbonates display no supercooling, sharp phase transitions, and high latent heats, reaching up to 227 J g^−1^ for tetradecyl carbonate. As renewable PCMs, they provide an alternative to paraffin wax.

It should be noted that processing compounds derived from biomass is not always environmentally sustainable. Some reactions require fossil fuel inputs or result in products with higher toxicity than their original precursors, such as the fatty amines derived from fatty acids. Due to their limited natural occurrence, we classified fatty amines as type 2 materials. These amines are typically synthesized by reacting ammonia with fatty acids at high temperatures in the presence of a metal oxide catalyst to give fatty nitriles, which are then hydrogenated using a Raney–Nickel catalyst.^[^ ^78^ ^]^ Fatty amine transition temperatures range from 26 °C for 1‐aminotridecane to 72 °C for dioctadecylamine, with a minimal supercooling effect of less than 10 °C.^[^ ^79^ ^]^ High enthalpies of fusion reaching 300 J g^−1^, despite a decrease after three cycles, remained nearly unchanged over the next 50 cycles, ranking fatty amines among the organic PCMs with the highest latent heat, comparable to that of sugar alcohols.

Recognizing the potential of amines, Poopalam et al. synthesized a series of monoamides and diamides. The monoamide *N*‐hexadecyl‐decanamide exhibited a melting point of 79 °C and an enthalpy of 201 J g^−1^.^[^ ^80^ ^]^ However, given the availability of more sustainable PCMs in this temperature range, more interesting are diamides with higher melting points >100 °C. Solvent and catalyst‐free high‐temperature (160–170 °C) condensation between C2–C10 diamines and C12–C18 fatty acids gave diamides with melting points in the intermediate temperature range (*T*_m_ of *N*,*N’*‐(octane‐1,8‐diyl)distearamide is 144 °C; Δ*H*_f_ = 218 J g^−1^) and remarkable stability over 100 heating and cooling cycles.^[^ ^81^ ^]^

Another group of PCMs for this temperature range are carbamates, synthesized by reacting diisocyanates with fatty alcohols.^[^ ^82^ ^]^ Some of the authors of this review recently demonstrated a series of carbamates, with dioctadecyl hexane‐1,6‐dicarbamate exhibiting a melting point of 121 °C and a high enthalpy of 200 J g^−1^, addressing the gap in PCMs with melting points above 100 °C. A drawback of this approach is the reliance on diisocyanates, the production of which involves phosgene. To mitigate the environmental impact, the authors explored an alternative synthetic route with the use of carbonates and fatty amines and analyzed the potential for product recycling and the costs of carbamate production, which accounted for $7.90 per kWh of stored energy per cycle.

Another group of versatile materials used in various PCM applications are polymers.^[^ ^83^ ^]^ Among them, HDPE stands out, with an impressive enthalpy of ≈230 J g^−1^, at a melting point of 130 °C, and stability over 500 heating and cooling cycles.^[^ ^84^ ^]^ Traditional HDPE production involves the polymerization of ethylene monomer using free radical or addition polymerization, often catalyzed by a Ziegler‐Natta catalyst, metal oxides, or metallocenes.^[^ ^85^ ^]^ To address sustainability concerns, efforts have shifted toward producing biobased polyethylene. Currently, biobased HDPE, accounting for 14% of the biobased bioplastic market, is primarily produced using sugarcane.^[^ ^86^ ^]^ This process involves dehydrating bioethanol derived from glucose to synthesize ethylene monomers, which are then polymerized using the same methods as fossil‐derived ethylene. The resulting biopolymer is chemically and mechanically identical to petroleum‐based PE, including its compatibility with recycling processes. Thus, biobased PE serves as a compelling example of a sustainable alternative to traditional polymer production while maintaining the desired properties for PCM applications.^[^ ^85^ ^]^

In the quest to develop materials with extended melting ranges for cold chain applications, lactones with valuable biological properties show significant promise. Two prominent lactones, γ‐valerolactone and ε‐caprolactone, stand out due to their low melting points, −32 and −1.55 °C, respectively, with enthalpies of fusion in the range of 100–120 J g^−1^.^[^ ^87^ ^]^ γ‐Valerolactone can be efficiently produced by catalytic hydrogenation of levulinic acid derived from cellulosic biomass, while ε‐caprolactone is typically synthesized on an industrial scale through the Baeyer–Villiger oxidation of cyclohexanone, where chemoenzymatic variations are possible.^[^ ^88^ ^]^ Therefore, despite the natural occurrence of many lactones, we classified them as type 2 bio‐PCMs. Despite their advantages, both compounds face limitations, including susceptibility to supercooling, limited stability over repeated thermal cycles, and potential degradation at elevated temperatures around 90–100 °C. These challenges impact their viability and highlight the need for further optimization to enhance their durability and stability for practical applications.

Research into efficient and sustainable production methods, such as enzyme‐ or microbial‐based transformations, is vital for advancing bio‐PCMs. Although some sustainably developed derivatives currently have less competitive thermal properties than well‐established fossil fuel‐derived PCMs, they still offer valuable insights into understanding the link between chemical structure and thermal properties. For type‐2 materials, which involve extensive chemical modifications, it is essential to balance thermal performance, economic feasibility, and environmental impact.

### 2.4 Type 3 Bio‐PCMs

As type 3 PCMs, we classified eutectic mixtures, uniform blends of two or more components (binary, ternary, or multicomponent systems) that melt within a narrow temperature range (**Table** **4**).^[^ ^89^ ^]^ This melting range is lower than the *T*_m_ of the individual components, making eutectic PCMs appropriate for creating materials with phase‐change temperatures that single‐component PCMs cannot achieve. The primary advantage of eutectic PCMs lies in their ease of preparation, as they can be simply created by combining different components, allowing for straightforward tuning of their melting temperature range, corrosivity, thermal conductivity, and stability.

**Table 4:** Thermal properties of TYPE 3 bio‐PCMs.^[^ ^250^, ^251^, ^252^, ^253^ ^]^

Type 3 bio‐PCMs					
Mixture of compound	*T*_m_ [°C]	Δ*H*_f_ [J g^−1^]	Mixture origin		References
			Biomass	Fossil fuels^a^^)^	
Lauric acid–myristic acid (0.85:0.15)	40	173	Fatty acids	–	[90, 250]
Myristic acid–stearic acid (0.50:0.50)	35–52	189			
1‐Dodecanol ‐ methyl stearate (0.10:0.90)	22	202	Fatty alcohol, fatty ester	–	[251]
1‐Dodecanol ‐ methyl palmitate (0.30:0.70)	20	224			
Paraffin‐stearic acid (0.45:0.55)	56.	192	Fatty acid	Paraffin	[252]
Paraffin wax‐ oleic acid	54	155	Fatty acid	Paraffin	[94]
Galactitol‐mannitol (0.30:0.70)	153	292	Sugar alcohol	–	[96, 97, 253]
Xylitol‐erythritol (0.64:0.36)	82	270	Sugar alcohol	–	
Erythritol‐xylitoll‐dulcitol (0.31:0.68:0.01)	81	268	Sugar alcohol	–	[97]
Stearic acid ‐ *n*‐butyramide (0.55:0.45)	64	198	Fatty acid	Amide	[95]
Succinic acid‐boric acid^b^^)^ (0.56:0.44)	150	394	Succinic acid	–	[17]

a) A compound derived from fossil fuels or produced through reaction with a fossil fuel‐based compound.

b) Boric acid is extracted from a variety of boron ores (colemanite, tincal, and ulexite) or naturally occurring boron brines.

Given the significant interest in using fatty acids as PCMs, it is unsurprising that much research focuses on developing their eutectic mixtures. PCMs with melting points in the low‐temperature range have been extensively studied for their application in construction materials aimed at thermal regulation in buildings.^[^ ^90^ ^]^ Fatty acid phase transition temperatures are typically too high to effectively regulate indoor environments, and therefore their eutectic mixtures have been developed to adjust to give thermal comfort in buildings. Sari^[^ ^90^ ^]^ successfully lowered the melting temperature of lauric acid and myristic acid (*T*_m_ = 43 and 52 °C respectively) to 34.2 °C by forming their eutectic mixture with a weight ratio of 66:34. The mixture exhibited a latent heat of 167 J g^−1^, and after 1460 thermal cycles, its value varied by only 2.4%, demonstrating potential for practical use.^[^ ^91^ ^]^ Gallart‐Sirvent et al. investigated direct preparation of PCMs from non‐edible fat waste through its hydrolysis followed by crystallization, yielding palmitic acid‐stearic acid mixtures.^[^ ^92^ ^]^ Depending upon the solvent used for the crystallization, the crystallized fractions revealed melting and freezing temperatures around 55 and 52 °C, respectively, while the latent heat values ranged from 172 to 181 J g^−1^.

Beyond using fatty acid eutectic mixtures, combinations of their esters were explored. Saeed et al.^[^ ^93^ ^]^ studied an eutectic mixture of methyl palmitate and lauric acid, which, at a molar ratio of 60/40, showed a high latent heat capacity (Δ*H*_m_ = 205.4) and phase change temperature (*T*_m_ = 25.6 °C) suitable for TES applications in buildings. The relatively low thermal conductivity of the mixture (0.1802 W m^−1^ K^−1^ in the solid state) could be enhanced by composite formation with carbon materials.

Recently, Chang et al. prepared eutectic mixtures of fatty acids (palmitic, stearic, and myristic acids) and paraffin wax with melting temperatures exceeding 55 °C and latent heat values up to 210 J g^−1^.^[^ ^94^ ^]^ As demonstrated by these authors, such PCMs are suitable for thermal management in electronic devices. To further expand the melting temperature range, Guixiang Ma et al. developed mixtures of stearic acid with *n*‐butyramide (*T*_m_ = 114 °C) and *n*‐octanamide (*T*_m_ = 107 °C). The resulting PCMs melted at 64 and 62 °C, respectively, with latent heat values of 198 J g^−1^ and excellent thermal stability over 100 cycles.^[^ ^95^ ^]^

Addressing the gap in medium‐temperature PCMs (*T*_m_ > 100 °C), Paul et al. investigated a PCM melting at 153 °C, prepared by mixing galactitol and mannitol at a molar ratio of 30:70.^[^ ^96^ ^]^ Moreover, the addition of small quantities (up to 0.5 wt%) of nucleating agents, such as graphite powder or silver iodide, to the PCM (which had a high latent heat of 292 J g^−1^) minimized its supercooling, additionally improving the latent heat value to 300 J g^−1^. Remarkably, after 100 thermal cycles, the latent heat decreased by only 4%. Sugar alcohol mixtures reveal promise for applications where melting points <100 °C are needed, as demonstrated for mixtures of xylitol and erythritol (*T*_m_ = 82 °C for 36 mol% erythritol) and mixtures of erythritol, xylitol, and dulcitol Ery(0.31)/Xyl(0.68)/Dul(0.01), *T*_m_ = 81 °C.^[^ ^97^ ^]^ However, the low stability under repetitive heating and cooling highlights the need for further research and modifications to enhance the performance of these bioderived systems.

A novel group of eutectic mixtures for intermediate TES was recently presented by Saher et al. with a record energy storage capacity of 394 J g^−1^.^[^ ^17^ ^]^ The eutectic mixture of boric acid and succinic acid undergoes a transition at around 150 °C, synergistically storing thermal energy by combining latent, thermochemical, and sensible heat. This opens new research directions for inexpensive and sustainable eutectic mixtures.

## 3 Biomass‐Derived Carbonaceous Supports for Shape‐Stabilized Phase‐Change Materials (SSPCMs)

As mentioned previously, PCMs have several disadvantages when it comes to application development, including leakage during the solid‐liquid phase transition process and poor thermal conductivity. To overcome these limitations, sustainable strategies have been explored to engineer SSPCMs. Besides direct incorporation and macro/microencapsulation in biopolymer matrices (see Section 4), the impregnation of PCMs into preformed, biomass‐derived porous supports has been seen as a cost‐effective alternative.^[^ ^98^, ^99^ ^]^ This approach usually involves a support soaked in a molten PCM or a dilute aqueous solution of PCM, the liquid being put into the support by vacuum impregnation. The support must, therefore, be sufficiently porous to absorb and retain as much PCM as possible. Several studies proposed the use of wood, previously delignified, to increase porosity and permeability.^[^ ^100^, ^101^ ^]^ Because wood is widely used in the field of building materials, it looks like the ideal candidate. Besides, if forests are managed responsibly and wood is manufactured or recycled in an eco‐friendly way, this resource is potentially sustainable. However, delignified wood or other lignocellulosic resources still have drawbacks for direct use in SSPCMs, such as low thermal conductivity, limited chemical stability (which will depend closely on the PCM used), high hygroscopic character, and poor control over porosity.

In addition to organic materials, nature provides inorganic and hybrid resources with remarkable properties. For instance, an original study proposed the use of artificially cultured diatom frustules,^[^ ^102^ ^]^ which come from biomass and display a hierarchical pore structure. However, diatom frustules are mainly composed of silica (80–90 wt%), which shows poor thermal conductivity and might adversely affect PCM performance. With this in mind, recent literature in the field has made it possible to draw up a set of specifications for defining appropriate support for SSPCMs. A suitable support should, therefore, be: 1) Renewable, sustainable, and low cost; 2) Highly porous, with an open pore network to allow high PCM loading and retention; 3) Chemically and thermally stable to ensure performance reliability over time; 4) Thermally conductive to sustain heat transfer and dissipation and improve TES efficiency; 5) Self‐standing and mechanically stable to make it easier to handle; 6) Sufficiently hydrophobic to avoid moisture adsorption/absorption; and optionally; 7) Electrically conductive if used in electrothermal PCM devices; 8) UV‐visible light absorber if used in photothermal PCM devices.

Among candidates that comply with all or most of these specifications, biomass‐derived carbonaceous supports present unique advantages.^[^ ^103^ ^]^ Polyaromatic and/or graphenic carbon materials can be prepared from a large variety of renewable, abundant, and low‐cost organic resources, such as lignocellulosic material and its derivatives. They are thermally conductive and chemically stable and offer tunable textural and structural properties. For these reasons, several studies have suggested their use in SSPCMs (**Table** **5**). For instance, G. Li et al. compared pristine poplar wood with the equivalent material pyrolyzed at 1000 °C under a nitrogen atmosphere.^[^ ^104^ ^]^ When used as supports for 1‐tetradecanol (1‐TDO), these authors reported higher 1‐TDO mass loading (73.4 vs. 60.1 wt%), higher latent heat of fusion (165.8 vs. 138.7 J g^−1^), and higher thermal conductivity (0.414 vs. 0.155 W m^−1^ K^−1^ at 25 °C) for pyrolyzed wood compared with pristine wood. This study, among others, confirms the potential of biomass‐derived carbonaceous materials in SSPCMs. We have selected about thirty recent (post‐2018) articles on this topic, which are discussed in the following.

**Table 5:** Summary of SSPCMs with biomass‐derived carbonaceous supports from articles published between 2018 and 2024.

Bioresource^a^^)^	Thermal treatment	PCM *|* *T*_m_ [°C]	Loading [wt%]	Melting enthalpy at 1st cycle [J g^−1^]	Melting enthalpy at *n* cycles | % retention	Thermal conductivity [W m^−1^ K^−1^] | increase [%]	Photothermal conversion efficiency [%]	References
Poplar wood	Washed in water	1‐TDO | 37.2	60.1	138.7	n/a	n/a	n/a	[104]
Poplar wood	1000 °C (N_2_)	1‐TDO | 37.2	73.4	165.8	200 | 84.4	0.52–0.66^a^ | 115.8–173.9	n/a	[104]
Cotton	MgO | 700 °C (Ar)	1‐HDO | 51.4	85.0	204.4	200 | 98.0	0.37^a^ | 5.7	n/a	[111]
Cotton	MgO | 800 °C (Ar)	1‐HDO | 51.4	90.0	219.4	200 | 95.4	0.40^a^ | 14.3	n/a	[111]
Cotton	MgO | 900 °C (Ar)	1‐HDO | 51.4	88.0	215.5	200 | 90.6	0.41^a^ | 17.1	n/a	[111]
Succulent	1000 °C (vacuum)	Paraffin | 40.8	62.8	83.6	n/a	n/a	82.0@300 mW cm^−2^	[119]
Succulent	1000 °C (vacuum)	Paraffin | 40.8	94.8	133.1	n/a	0.31^a^ | 25.0	n/a	[119]
Fresh potato	1300 °C (N_2_)	PEG 4000 | 58.8	85.4	158.8	n/a	4.49^b^ | 951.0	n/a	[110]
White radish	1300 °C (N_2_)	PEG 4000 | 58.8	89.8	159.7	n/a	1.75^b^ | 309.8	n/a	[110]
Sunflower straw	400 °C	Stearic acid | 68.1	83.7	186.1	n/a	0.33^c^ | 106.3	n/a	[254]
Sycamore wood	Delignified 500 °C (N_2_)	Lauric acid | 42.8	81.1	177.9	100 | 98.8	0.270^ d^ | n/a	n/a	[255]
Cotton	1200 °C (Ar) Pressed at 16 mm	Paraffin | 25.5	95.5	209.3	n/a	0.29^c^ | 17.6	n/a	[112]
Cotton	2400 °C (Ar) Pressed at 10 mm	Paraffin | 25.5	92.2	202.2	n/a	0.43^c^ | 73.6	n/a	[112]
Cotton	2400 °C (Ar) Pressed at 16 mm	Paraffin | 25.5	95.1	205.7	n/a	0.36^c^ | 43.2	n/a	[112]
Cotton	900 °C (Ar)	Paraffin | 40.2	84.1	182.0	n/a	0.40^e^ | 39.9	76.4@100 mW cm^−2^	[120]
Sunflower stem	1000 °C (Ar)	1‐HDA | 44.0	95.2	271.0	50 | 99.7	0.38^f^ | 157.8	67.8@100 mW cm^−2^	[117]
Sunflower stem	1000 °C (Ar)	Palmitic acid | 61.8	n/a	233.8	50 | 92.6	0.34^f^ | 111.1	n/a	[117]
Sunflower rec.	1000 °C (Ar)	1‐HDA | 44.0	89.7	239.4	50 | 99.7	0.37^f^ | 153.7	75.6@100 mW cm^−2^	[117]
Sisal fibers	2400 °C (Ar) Density 0.097 g cm^−3^	Paraffin | 57.8	91.3	201.6	100 | 99.9	0.42–0.95^f^ | 68–280	n/a	[127]
Sisal fibers	2400 °C (Ar) Density 0.172 g cm^−3^	Paraffin | 57.8	87.2	192.2	100 | 99.9	0.62–1.73^f^ | 148–592	n/a	[127]
Sucrose	NaHCO_3_ | 800 °C (N_2_)	Paraffin | 25.4	84.0	152.2	1000 | 100.5	0.33^a^ | 22.2	31.0@200 mW cm^−2^ 89.0@300 mW cm^−2^	[123]
Towel gourd	900 °C (N_2_)	PEG 2000 | 58.9	94.5	164.3	n/a	n/a	n/a	[129]
Watermelon	HTC 200 °C	Mannitol | 170.2	97.0	281.9	n/a	0.78^ g^ | 20	n/a	[118]
Watermelon	HTC 200 °C	Mannitol | 170.2	88.0	255.5	n/a	1.05^ g^ | 61.5	n/a	[118]
Gelatin	Fe(NO_3_)_3_ | 800 °C (N_2_)	Eicosane | 35.8	85.8	211.7	1000 | 96.6	0.52^f^ | 13	60.6@200 mW cm^−2^ 93.3@300 mW cm^−2^	[122]
Water chesnut	HTC 130 °C AlO_6_P_3_ | 800 °C (N_2_)	Octadecane | 29.7	85.0	216.2	100 | 99.0	1.47^a^ | 673	96.1@100 mW cm^−2^	[133]
Water chesnut	HTC 130 °C AlO_6_P_3_ | 900 °C (N_2_)	Octadecane | 29.7	80.0	192.3	n/a	1.52^a^ | 700	n/a	[133]
Balsa	Delignified	Paraffin | 41.5	85.1	199.9	30 | 86.5	n/a	60.0@100 mW cm^−2^	[135]
Balsa	Delign. | 300 °C (N_2_)	Paraffin | 41.5	85.0	201.7–206.3	30 | 91.7–95.7	0.135–0.182^h^ | n/a	85.6–90@100 mW cm^−2^	[135]
Corn straw	400 °C (N_2_)	PEG | 56.8	36.8	105.4	n/a	0.190^h^ | n/a	49.3@100 mW cm^−2^	[107]
Corn straw	600 °C (N_2_)	PEG | 56.8	48.4	100.2	100 | 98.7	0.330^h^ | 1.9	69.0@100 mW cm^−2^	[107]
Corn straw	800 °C (N_2_)	PEG | 56.8	42.6	77.2	n/a	0.429^ h^ | 32.4	55.6@100 mW cm^−2^	[107]
Pomelo peel	900 °C (N_2_)	PEG 4000 | 61.3	95.4	162.4	n/a	n/a	84.0@200 mW cm^−2^	[121]
Pomelo peel	Freeze‐dried	Paraffin | 59.5	88.0	125.7	25 | 100.7	0.218^a^ | 6.3	n/a	[106]
Pomelo peel	650 °C (Ar)	Paraffin | 59.5	93.4	158.6	25 | 100.3	0.207^a^ | 1.0	60.1@200 mW cm^−2^	[106]
Pomelo peel	750 °C (Ar)	Paraffin | 59.5	94.2	159.5	25 | 100.3	0.300^a^ | 46.3	85.1@200 mW cm^−2^	[106]
Pomelo peel	850 °C (Ar)	Paraffin | 59.5	95.6	150.3	25 | 99.7	0.312^a^ | 52.2	70.4@200 mW cm^−2^	[106]
Bamboo	Dried	Paraffin | 28.9	n/a	99.8	100 | 98.8	0.28^a^ | 17.3	n/a	[256]
Bamboo	600 °C (N_2_)	Paraffin | 28.9	n/a	79.4	100 | 101.0	0.42^a^ | 72.4	n/a	[256]
Bamboo	H_3_PO_4_ | 600 °C (N_2_)	Paraffin | 28.9	n/a	59.5	100 | 99.3	0.52^a^ | 116.2	n/a	[256]
Rice husk	K_2_CO_3_ | 700 °C (N_2_)	Stearic acid | 70.9	80.3	162.1	n/a	0.438^i^ | 13.8	n/a	[108]
Bamboo	K_2_CO_3_ | 700 °C (N_2_)	Stearic acid | 70.9	79.4	159.0	n/a	0.405^i^ | 5.2	n/a	[108]
Pine wood	K_2_CO_3_ | 700 °C (N_2_)	Stearic acid | 70.9	82.9	170.6	n/a	0.393^i^ | 2.1	n/a	[108]
Walnut shell	K_2_CO_3_ | 700 °C (N_2_)	Stearic acid | 70.9	78.5	168.9	n/a	0.418^i^ | 8.6	n/a	[108]
Corncob	K_2_CO_3_ | 700 °C (N_2_)	Stearic acid | 70.9	80.8	175.3	n/a	0.389^i^ | 1.0	n/a	[108]
Bamboo	Ca(OH)_2_ | 550 °C (Ar)	1‐ODO | 59.0	70.0	197.0	n/a	0.71^j^ | 184	75.2@300 mW cm^−2^	[124]
Bamboo	Ca(OH)_2_ | 550 °C (Ar)	1‐ODO | 59.0	80.0.	284.1	n/a	0.57^j^ | 128	66.4@300 mW cm^−2^	[124]
Bamboo	Ca(OH)_2_ | 550 °C (Ar)	1‐ODO | 59.0	85.0	299.8	n/a	0.45^j^ | 80	57.3@300 mW cm^−2^	[124]
Sunflower stem	1000 °C (N_2_)	PEG 4000 | 62.5	84.0	153.4	50 | 99.6	0.43^a^ | 51.9	n/a	[125]
Sunflower rec.	1000 °C (N_2_)	PEG 4000 | 62.5	91.6	171.5	50 | 99.4	0.41^a^ | 44.9	n/a	[125]
Loofah sponge	KOH | 800 °C	Octadecane | n/a	60.4	155.1	100 | 99.2	0.208^a^ | 27.7	95.2@100 mW cm^−2^	[136]
Sorghum straw	Delignified HTC 220 °C CQDs	PEG 2000 | 53.1	94.5	168.1	100 | 93.8	0.374^a^ | n/a	90.8@1 W cm^−2^	[109]

a) All data is adopted from the literature; *T*_m_: melting point; Sunflower rec.: Sunflower receptacle; 1‐TDO: 1‐tetradecanol; 1‐HDO: 1‐hexadecanol; 1‐HAD: 1‐hexadecanamine; 1‐ODO: 1‐octadecanol. Methods and instruments used to measure thermal conductivities: ^a^transient plane source method (TPS 2500S or 2500‐OT, Hot Disk instruments), ^b^xenon flash method (XFA 500, LINSEIS), ^c^laser flash method (undefined), ^d^laser flash method (LFA 457, NETZSCH), ^e^transient plane source method (TPS, DRE‐III tester, XIANGTANXIANGYI), ^f^n/a, ^g^transient plane source method (HS‐DR‐5, HESON), ^h^laser flash method (LFA 467, NETZSCH); ^i^transient plane source method (TCi, C‐Therm), ^j^transient plane source method (TPS 3500S, Hot Disk instruments).

### 3.1 Sustainability Considerations

If they come from a renewable, readily available, and inexpensive resource, which is not in competition with the food industry, carbonaceous supports can be considered sustainable. In the literature, several bioresources and agrowastes have been proposed as precursors to carbonaceous supports for SSPCMs, such as garlic peel,^[^ ^105^ ^]^ pomelo peel,^[^ ^106^ ^]^ corn straw,^[^ ^107^ ^]^ rice husk, pine wood,^[^ ^108^ ^]^ and sorghum straw.^[^ ^109^ ^]^ Although the majority of these carbon precursors were agricultural or food waste with no significant added value, the question of their availability and sourcing was not addressed. Some precursors are even food resources, such as fresh potato and white radish,^[^ ^110^ ^]^ or already have an existing market with added value, such as cotton.^[^ ^111^, ^112^ ^]^ Apart from being renewable and low cost, the production and storage of biomass‐derived carbons in buildings and structures has been proposed as a means of mitigating climate change;^[^ ^113^ ^]^ indeed, they sequester carbon for a much longer period of time than would be the case if the original biomass were left to decay, following the example of biochars in soils.^[^ ^114^ ^]^ Interestingly, the use of carbonaceous materials that do not come from bioresources but are part of a circular economy can also be a relevant option when it comes to sustainability. For instance, Z. Huang et al. proposed the use of graphite extracted from spent lithium‐ion batteries to prepare SSPCMs with excellent solar‐thermal and electric‐thermal conversion properties.^[^ ^115^ ^]^

Another important feature to take into account when dealing with biomass‐derived carbonaceous supports is the thermochemical process used to convert organic precursors into porous carbon‐rich materials. Whether pyrolysis under an inert atmosphere or chemical activation is applied, the authors rarely assess the impact of the chemical process through the prism of green chemistry precepts.^[^ ^116^ ^]^ Carbonization yields are often very low (below 20 wt%), and chemical activation frequently involves harmful chemicals and generates a large amount of waste, leading to a high E‐factor. Nonetheless, sustainable alternatives are available. For instance, W. Ma et al. replaced KOH, a chemical activating agent that is highly corrosive, with potassium carbonate to prepare carbon‐based SSPCMs.^[^ ^108^ ^]^ In general, lifecycle and techno‐economic analyses will have to be carried out to select the most sustainable resources and carbonization processes. Last, but not least, there are very few examples of SSPCMs with fully biomass‐derived constituent materials, that is, both carbon support and PCM;^[^ ^108^, ^117^ ^]^ however, fully biobased SSPCMs with phase change enthalpies of up to 234 and 282 J g^−1^ were reported for palmitic acid in sunflower‐derived carbon^[^ ^117^ ^]^ and mannitol in watermelon‐derived carbon,^[^ ^118^ ^]^ respectively. As a result, this review should open up many new avenues for future research studies.

### 3.2 Influence of the Porosity and Texture of the Carbon Support

High porosity, with an open pore network, is mandatory to allow high PCM loading and retention over time. Logically, the higher the porosity, the larger the PCM loading, and the higher the phase‐change enthalpy should be.^[^ ^119^, ^120^ ^]^ Recent articles reported PCM loading capacities superior to 95 wt%^[^ ^106^, ^112^, ^117^, ^118^, ^121^ ^]^ and retention of the phase‐change enthalpy superior to 96% over 1000 cycles^[^ ^122^, ^123^ ^]^ for SSPCMs based on biomass‐derived carbonaceous supports. Coincidently, the larger the PCM loading, the lower the thermal conductivity of the SSPCM.^[^ ^120^, ^123^, ^124^ ^]^ Controlling the structure, texture, and porosity of the carbon support should allow for a good compromise between those properties.

Although macropores (diameter >50 nm) are inherent to the selected biomass and can be modulated with difficulty (e.g., by delignification), micropores (<2 nm) and mesopores (between 2 and 50 nm) can be developed by a judicious choice and combination of thermochemical treatments and/or templates.^[^ ^111^ ^]^ Biomass‐derived carbonaceous materials with various specific surface areas (up to 1640 m^2^ g−1^[^ ^125^ ^]^), pore size distributions, and pore volumes have been tested in SSPCMs. However, textural characterizations are often limited to volumetric gas adsorption and electron microscopy. Therefore, a global view of porosity, with a quantification of each contribution from macropores to ultramicropores (below 0.7 nm), is often lacking. Moreover, it is difficult, if not impossible, to study the influence of porosity independently of other parameters. Indeed, for the same biomass, controlling porosity is often associated with modifying the temperature of the thermochemical treatment and/or adding activating agents, which can considerably modify the surface chemistry and/or the structure of the carbonaceous framework.

Although the role of porosity and texture of the carbon support is difficult to assess, a few studies have attempted to tackle this important question. X. Li et al.^[^ ^126^ ^]^ studied the phase change behavior of PEG 6000 impregnated into three different types of carbon materials: expanded graphite (EGr) bearing only macropores centered at 10 μm, an activated carbon (AC, obtained by physical steam activation of coconut shell) bearing mainly micropores, and an ordered mesoporous carbon (CMK‐5, obtained from sucrose as the carbon source) bearing both micro‐ and mesopores. The authors concluded that the macropores in EGr allowed a higher relative enthalpy efficiency and, consequently, a higher heat storage capacity to be maintained, whatever the mass loading of PEG (from 60 to 90 wt%) in the SSPCM. The melting enthalpy of EGr‐SSPCM, therefore, remained above 100 J g^−1^ even with 60 wt% of PEG. In this study, the authors also concluded that the macropores should be neither too wide, so that capillary forces are sufficiently high to retain the PCM in the composite, nor too narrow, so as not to affect the PCM crystallinity and heat storage capacity of the composite. Nevertheless, these results must be treated with caution. As shown by powder X‐ray diffraction, the structure of EGr is graphitic, while both AC and CMK‐5 display turbostratic structures. Besides, the surface chemistry of the carbon materials and their chemical interaction with the PCM were not thoroughly studied and discussed.

In addition to porosity, density is another parameter to be considered. In two different studies, N. Sheng et al. prepared carbon supports with adjustable density derived from cotton^[^ ^112^ ^]^ and sisal fibers^[^ ^127^ ^]^ to study the phase change behavior of paraffin. In both studies, the authors showed that, for the same specific surface area and micro‐mesopore volume, the SSPCMs with the densest carbon framework had the lowest paraffin mass loadings and the lowest phase change enthalpies (Table 5). In contrast, the densest supports had higher thermal conductivities and comparable relative enthalpy efficiencies. In these two studies, density was directly related to the volume of the interstices between the fibers and, therefore, to the volume of the largest pores, demonstrating the predominant influence of macropores on the properties of SSPCMs. At this point, it is important to note that, after thermal posttreatment, whether pyrolysis or activation, the porosity (micro‐ and mesopore volumes) generated in the carbonaceous framework significantly lowers both the envelope and bulk density of the material (since inaccessible narrow pores and closed pores are often produced). At constant volume, the PCM mass loading can, therefore, increase considerably without this effect being directly attributable to the interactions of the PCM with the micro‐ and mesopores themselves.^[^ ^128^, ^129^ ^]^

**Figure 3:**
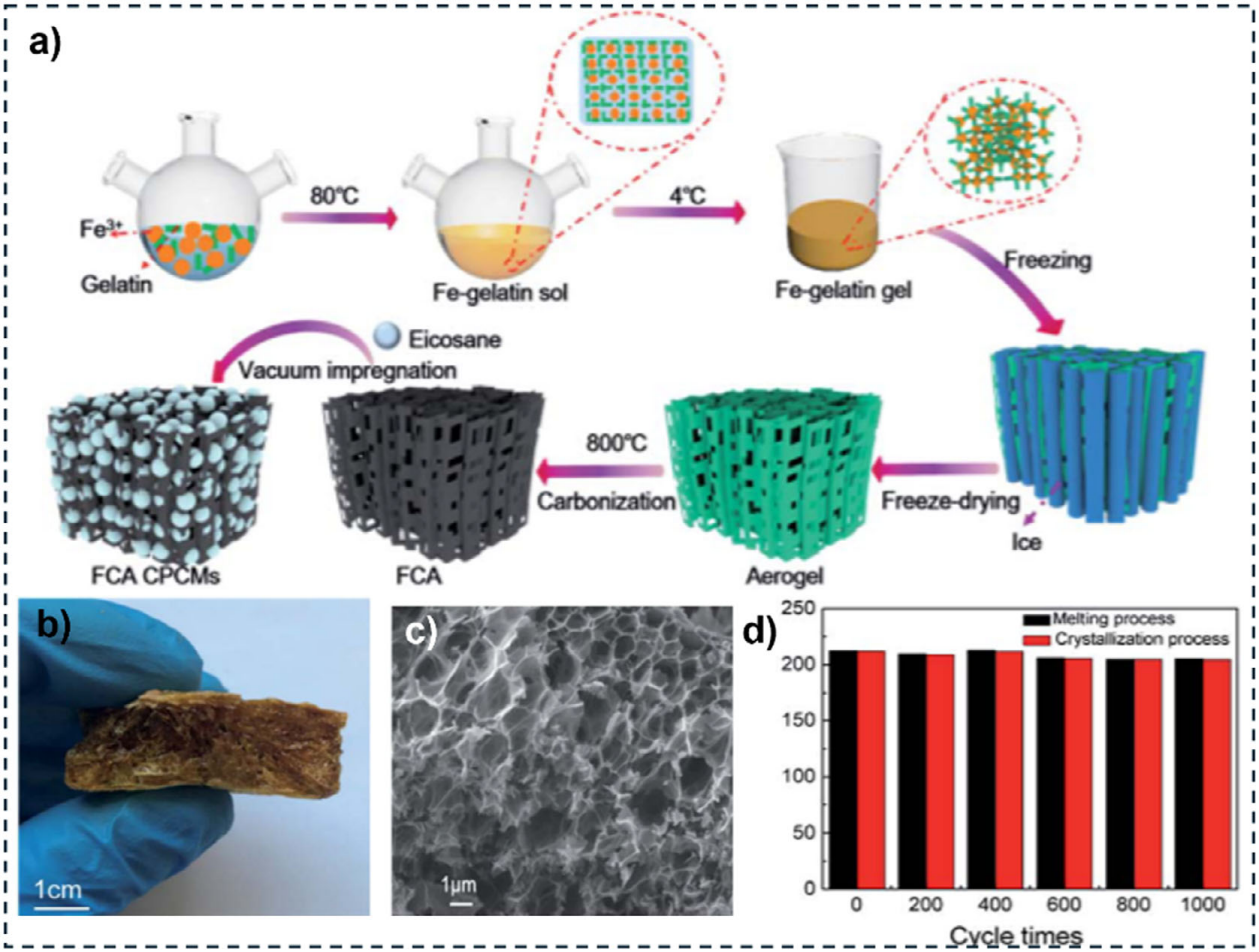
a) Preparation method for gelatin‐derived carbon aerogel composite. b) Aerogel after freeze‐drying treatment before the carbonization process. c) Scanning electron microscopy (SEM) image after the carbonization process. d) Stability of the composite over 1000 cycles. Reproduced with permission.^[^ ^122^ ^]^ Copyright 2021, Royal Society of Chemistry.

It is also important not to overlook the effects of confinement in micro‐mesopores on phase transitions of PCMs that may result in significant shifts, especially when interfacial interactions with the pore walls are stronger than intermolecular interactions between the neighboring molecules confined in the pores.^[^ ^130^, ^131^ ^]^ For instance, G. Wang et al. showed that cotton‐derived porous carbons with specific surface areas between 490 and 880 m^2^ g^−1^ favorably lowered the extent of the supercooling phenomenon of pristine 1‐hexadecanol by 20.9–26.4%.^[^ ^111^ ^]^ Q. Shi et al. reported a decrease in the supercooling extent of eicosane by ≈64.2% by confinement in a gelatin‐derived carbon (**Figure** **3**) that displayed a specific surface area of 250 m^2^ g^−1^.^[^ ^122^ ^]^ More recently, X. Liu et al. reported a decrease in the supercooling extent of PEG 6000 by ≈46.2% by confinement in sunflower‐derived highly porous carbons (>1000 m^2^ g^−1^).^[^ ^125^ ^]^ In these examples, porosity seems to play a major role in the shift of the phase transition temperatures. Additionally, the molecular motion of PCM can be considerably hindered by confinement in narrow pores, therefore decreasing phase‐change enthalpy and heat storage capacity. A thorough study of the influence of porosity independently of other parameters would require model carbonaceous materials with a multiscale control over textural properties (e.g., pore size distribution, roughness, shape, hierarchy, and connectivity).

### 3.3 Surface Chemistry and Interfacial Interactions with the PCM

Interfacial interactions closely depend on the chemistry on the pore walls. Due to a PCM's confinement in narrow pores, the surface chemistry can have a major impact on its molecular motion. Nevertheless, in the context of SSPCMs, the surface chemistry of the carbon support and its interaction with PCMs have been little studied. On the one hand, strong interactions between the PCMs and pore walls would have a major impact on both the supercooling phenomenon (i.e., lower melting point and higher crystallization temperature) and the phase‐change enthalpy (decrease in heat storage capacity). On the other hand, it can be assumed that high‐temperature pyrolysis can promote hydrophobic carbon surfaces that may limit the ads‐/absorption of water molecules both on these surfaces and on the PCM. Interestingly, G. Li et al. demonstrated that delignified wood coated with a superhydrophobic surface prevented liquid leakage, brought self‐cleaning functionality to the composite, and significantly improved heat storage capacity in humid environments.^[^ ^101^ ^]^ These authors reported a latent heat of fusion of 125.4 J g^−1^, which was 29.6 J g^−1^ higher than that of the phase change composite without the superhydrophobic coating. Such an approach remains to be demonstrated for biomass‐derived carbonaceous materials.

### 3.4 Shape Stabilization and Leakage Protection

Shape stabilization and leakage protection were systematically investigated (apart from a few exceptions) by observation with a digital camera above the melting point of PCM. In most cases, a slight liquid leakage was observed, which was attributed to the thermal expansion of the PCM during the melting process. Leakage measurements were, however, rarely performed over a period of more than 2 h,^[^ ^110^, ^118^, ^119^, ^123^, ^129^ ^]^ and the mass losses of the PCM were rarely quantified. Furthermore, PCM leakage during heating‐cooling cycles was rarely, if ever, investigated. In two of the rare examples, PCM leakage of less than 3 wt% was observed after 100^[^ ^125^ ^]^ and 200 thermal cycles^[^ ^121^ ^]^ for PEG entrapped in sunflower‐ and pomelo peel‐derived porous carbons, respectively. Both parameters, that is, shape stabilization and leakage protection, should be investigated in more detail in order to comprehensively evaluate the potential integration of SSPCMs in TES systems.

### 3.5 Factors Affecting Thermal and Electrical Conductivity

As mentioned above, high thermal conductivity is mandatory to sustain heat transfer and dissipation and improve TES efficiency. We have already discussed two important and directly related parameters: the PCM loading and the density of the carbon support. On the one hand, as the PCM loading increases, the thermal conductivity of the SSPCM decreases.^[^ ^120^, ^123^, ^124^ ^]^ On the other hand, SSPCMs with the densest carbon supports generally have higher thermal conductivities.^[^ ^112^, ^127^ ^]^ Two other important parameters can affect both thermal and electrical conductivities: the nanotexture (the dimensions of the crystallites and the presence of defects) and the structure (amorphous, turbostratic, partially graphitic, or graphitic) of the carbon support. Depending on the carbon precursor and on the thermochemical process used to convert it into a graphenic carbon‐rich material, these two parameters can be significantly affected. In general, as the pyrolysis temperature increases, defects and distortions within the graphene layer ensembles are healed. Coincidently, the structure evolves from amorphous to turbostratic and then from turbostratic to graphitic (beyond 2800 °C). Both phonons and electrons experience stronger scattering at lattice defects, chemical impurities, and grain boundaries. Thus, the higher the defect level, the shorter the mean free path of both phonons and electrons, and the lower the thermal and electrical conductivities.^[^ ^132^ ^]^ Consequently, as the pyrolysis temperature increases, both the thermal and electrical conductivities should increase. This trend was confirmed in SSPCMs with carbon supports derived from cotton,^[^ ^111^, ^112^ ^]^ corn straw,^[^ ^107^ ^]^ and waste pomelo peels.^[^ ^106^ ^]^ However, it should be noted that it is difficult, if not impossible, to study the influence of one parameter independently of the others.

Interestingly, most studies using biomass‐derived carbon supports in SSPCMs demonstrated significant improvement in thermal conductivity compared to pristine PCMs (Table 5). For instance, Sheng et al. reported an improvement of 592% and a value of 1.73 W m^−1^ K^−1^ for sisal‐derived carbon fibers loaded with 87.2 wt% paraffin.^[^ ^127^ ^]^ In another study, Zhao et al. showed an improvement of 700% and a value of 1.52 W m^−1^ K^−1^ for water chestnut‐derived carbons loaded with 80 wt% of octadecane.^[^ ^133^ ^]^ The most significant improvement was reported by Y. Zhao et al. with potato‐derived carbon loaded with 85.4 wt% of PEG 4000. The authors showed a thermal conductivity of 4.49 W m^−1^ K^−1^, corresponding to 1050% of the value for the pristine PCM.^[^ ^110^ ^]^

Thermal conductivity can be further increased by adding inorganic fillers, which are not necessarily derived from biomass. For instance, Xie et al. demonstrated that the addition of silver nanoparticles in grapefruit peel‐derived porous carbon allowed a twofold increase in thermal conductivity compared to silver‐free SSPCMs and a threefold increase compared to the pristine PCM.^[^ ^134^ ^]^

### 3.6 Photothermal and Electrothermal Conversion Efficiency

When it comes to applications, an important feature is the ability of SSPCMs to convert either light or electricity into thermal energy. Photothermal and electrothermal conversion efficiencies are not only directly related to the thermal and electrical conductivities mentioned above but also to the UV‐visible light absorption capacity. While most PCMs show relatively low absorption of solar energy, polyaromatic and graphenic carbon materials display high UV‐visible light absorption capacity.^[^ ^135^ ^]^ For instance, J. Ren et al. prepared carbon quantum dots (CQDs) by hydrothermal carbonization of lignin (extracted from sorghum straw) to put inside delignified sorghum straw along with PEG 2000.^[^ ^109^ ^]^ It was possible to reach a photothermal conversion efficiency of 90.8% using a 750 nm laser with a constant intensity of 1 W cm^−2^. The phase transition temperature of PEG 2000 inside delignified sorghum straw without CQDs was not reached in similar photothermal experiments, demonstrating the importance of using carbonaceous supports with high light absorption capacity. Recent articles showed high photothermal conversion efficiencies of more than 75% under a light intensity of 100 mW cm^−2^, together with a high phase‐change enthalpy above 150 J g^−1^ (Table 5).^[^ ^117^, ^133^, ^135^, ^136^ ^]^ For instance, P.‐P. Zhao et al. reported a photothermal conversion efficiency of 96.1% (@100 mW cm^−2^) and a phase‐change enthalpy of 216.2 J g^−1^ with water chestnut‐derived carbons loaded with 85 wt% of octadecane.^[^ ^133^ ^]^

**Figure 4:**
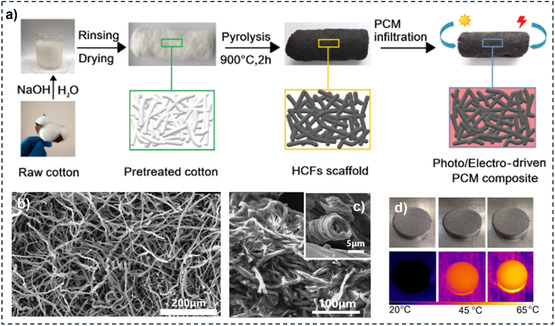
a) Preparation method for cotton‐derived carbon composites. b) SEM image of the carbon scaffold. c) SEM image of the composite. d) Form stability and heat distribution performance of the composite. Reprinted with permission.^[^ ^120^ ^]^ Copyright 2020, American Chemical Society.

The electrothermal conversion ability of biomass‐derived carbonaceous supports for SSPCMs has been less frequently reported in the literature. In one of the rare examples, M. M. Umair et al. showed a high phase‐change enthalpy of 182.2 J g^−1^ together with high photothermal (76.4%@100 mW cm^−2^) and electrothermal conversion efficiencies for cotton‐derived carbon loaded with 84.1 wt% of paraffin (**Figure** **4**).^[^ ^120^ ^]^ This composite had a very high electrical conductivity of 19.6 S m^−1^, versus 10^−14^ S m^−1^ for pristine paraffin. The authors reported an electrothermal storage efficiency of 81.1% under 3 V, which was significantly better than the one of 71.4% under 15 V previously reported by Y. Li et al.^[^ ^137^ ^]^ for melon‐derived carbon loaded with paraffin.

In conclusion, the use of biomass‐derived carbonaceous materials in shape‐stabilized phase‐change composites looks very promising as these materials comply with all the specifications that define appropriate supports for SSPCMs. Biomass‐derived carbonaceous materials are renewable, low‐cost, chemically stable, and thermally and electrically conductive. Moreover, they display high UV‐visible light absorption capacity and offer tunable textural and structural properties. Consequently, these supports could give a large PCM loading (above 95 wt%), high phase‐change enthalpy (above 250 J g^−1^), and good retention of the phase‐change enthalpy over time (up to 100% over 1000 cycles; Table 5). Moreover, biomass‐derived carbonaceous supports allowed competitive photothermal and electrothermal conversion efficiencies of 96.1 (@100 mW cm^−2^)^[^ ^133^ ^]^ and 81.1% (@3 V),^[^ ^120^ ^]^ respectively, to be reached. However, most studies have focused on the performance of SSPCMs but provide little insight into understanding the interaction between the PCM and the carbonaceous support. It should be noted that it is difficult, if not impossible, to study the influence of one parameter independently of the others. When it comes to wood and raw lignocellulosic biomass, certain properties are difficult to control and modulate. For instance, macroporosity is inherent to the selected bioresource and might be significantly affected by seasonal variability. A thorough investigation of the influence of porosity, nanotexture, structure, and surface chemistry would require model materials where all these properties can be precisely controlled. The contribution of molecular dynamics simulation and machine learning could also be beneficial. Finally, yet importantly, lifecycle and techno‐economic analyses will have to be carried out to select the most sustainable resources and carbonization processes.

## 4 Purpose of Encapsulation of PCMs

The encapsulation of PCMs addresses key performance challenges in LHS systems. In particular, encapsulation serves as an effective approach to improve the thermal conductivity of PCMs by increasing the surface area available for heat transfer, thereby enhancing the overall performance of thermal storage applications.^[^ ^138^, ^139^, ^140^, ^141^ ^]^ In addition to improved thermal conductivity, encapsulation provides a robust barrier that protects PCMs from environmental factors, such as moisture and oxygen, which could otherwise lead to material degradation by corrosion or oxidation, leading to a loss of efficiency over time.^[^ ^138^ ^]^

Finally, encapsulation eliminates issues such as leakage, a significant concern during phase transitions when PCMs shift from a solid to a liquid. By containing the PCM within a stable shell, encapsulation prevents the material from escaping, therefore retaining its effectiveness and extending its operational life.^[^ ^142^, ^143^ ^]^

The encapsulation process mimics nature's strategies, being similar to the protective structures found in cells and eggshells, with the PCM acting as the core material and the encapsulation material forming a surrounding shell.^[^ ^29^, ^30^ ^]^ This design concept allows for flexibility in the selection of encapsulating materials, enabling researchers to tailor the shell's properties to meet specific needs. Typically, shell materials fall into three primary categories: organic, inorganic, and hybrid materials. Organic shells, often polymer‐based, offer chemical compatibility with organic PCMs and can be designed to enhance flexibility and impact resistance. In contrast, inorganic shells may provide superior thermal stability and mechanical strength, suitable for PCMs used in higher‐temperature applications. Hybrid shells combine organic and inorganic materials to leverage the advantages of both, resulting in encapsulated PCMs with enhanced thermal conductivity, durability, and chemical compatibility. Selecting an appropriate shell material is crucial and hinges on factors such as interfacial properties and the intermolecular forces between the core PCM and shell. These properties influence the stability, strength, and thermal conductivity of the encapsulated PCM, which are key parameters for optimizing performance in specific applications. Importantly, the shell must not only encapsulate the PCM effectively but also withstand repeated thermal cycling, maintain structural integrity, and, ideally, enhance the thermal conductivity of the PCM system. Thus, encapsulation techniques not only safeguard PCMs from environmental degradation and leakage but also enable a customizable approach to improve thermal conductivity, mechanical strength, and overall compatibility with a variety of storage materials. This adaptability supports the development of LHS systems optimized for diverse applications, from medicine^[^ ^144^ ^]^ to high‐temperature industrial uses.

The use of renewable, biodegradable materials, particularly biopolymers, offers a promising solution for creating eco‐friendly encapsulation systems that reduce the environmental impact associated with traditional, synthetic encapsulating materials.^[^ ^33^, ^145^ ^]^ Biopolymers, derived from natural sources, including agricultural waste and the food industry, can provide a renewable alternative that minimizes environmental pollution. Additionally, biopolymers are inherently biocompatible, making them suitable for applications in environmentally sensitive fields, such as food packaging,^[^ ^146^ ^]^ textiles,^[^ ^147^ ^]^ and medical devices.^[^ ^148^ ^]^ By harnessing these renewable sources, researchers can develop encapsulation materials that not only enhance PCM performance through improved thermal stability and mechanical properties but also support sustainable development and circular economy principles, ensuring that advances in TES align with broader environmental sustainability goals.^[^ ^149^, ^150^ ^]^

Encapsulation of PCMs can be categorized into three main types based on the scale of the encapsulated particles: nano‐, micro‐, and macroencapsulation.

1) **Nanoencapsulation** creates extremely small particles, typically under 100 nm in diameter. Nanoencapsulation can significantly increase the surface area‐to‐volume ratio, providing rapid heat transfer, controlled release, and excellent containment of the PCM. For biopolymer‐based encapsulation, nanoencapsulation offers the potential to improve the thermal conductivity and mechanical strength of PCMs while using a minimal amount of material. Nanoencapsulated PCMs are used in fields that require precise thermal control and high‐performance thermal conductivity, such as electronics, medical devices, and advanced textiles. Nanoencapsulation with biopolymers is still in its early stages, but it holds potential for high‐efficiency thermal management in applications where rapid phase transition and compact size are critical. For a better understanding, please refer to Shchukina et al. 2018.^[^ ^151^ ^]^

2) **Microencapsulation** involves creating capsules with diameters ranging from 1 to 1000 μm. This technique is widely used for PCMs in various applications due to the balance it offers between surface area and structural integrity. Microencapsulation with biopolymers is particularly popular for PCMs in textiles, construction materials, and energy storage, where controlled heat release and durability are essential.^[^ ^152^ ^]^

3) **Macroencapsulation** refers to encapsulation techniques that create capsules larger than 1 mm in diameter, often reaching several centimeters. Macroencapsulation is valuable when larger volumes of PCM are needed for high‐capacity energy storage applications, such as building insulation and industrial heat management.^[^ ^153^ ^]^

### 4.1 Biopolymers as Encapsulation Materials

Biopolymers, derived from renewable sources, have emerged as promising encapsulation materials, providing a sustainable and biodegradable alternative to traditional synthetic polymers. Unlike synthetic polymers, which often rely on petroleum‐based sources, biopolymers offer environmental benefits and unique properties that make them particularly advantageous for PCM encapsulation. This section explores the critical role of biopolymers in PCM encapsulation, highlights their advantages over synthetic polymers, and discusses the most commonly used biopolymers for this purpose.

Chitosan (CS) is a biopolymer derived from chitin, which is found primarily in the exoskeletons of crustaceans such as shrimp and crabs,^[^ ^154^, ^155^ ^]^ making it a renewable and biodegradable material ideal for PCM encapsulation applications.^[^ ^156^ ^]^ In recent years, CS has garnered considerable interest as an encapsulating agent for PCMs, mostly due to its sustainable origin, environmental compatibility, and unique film‐forming capability, which provides the structural integrity essential for encapsulating PCMs effectively by preventing leakage during phase transitions. This ability to create stable shells enhances the performance and lifecycle of PCM‐encapsulated systems that rely on multiple heating and cooling cycles. As a biodegradable material, CS addresses the need for eco‐friendly materials in applications where environmental impact is of concern. Furthermore, CS's antimicrobial properties inhibit microbial growth, which is advantageous in applications such as food packaging and biomedical fields, where product safety and resistance to contamination are critical.

**Figure 5:**
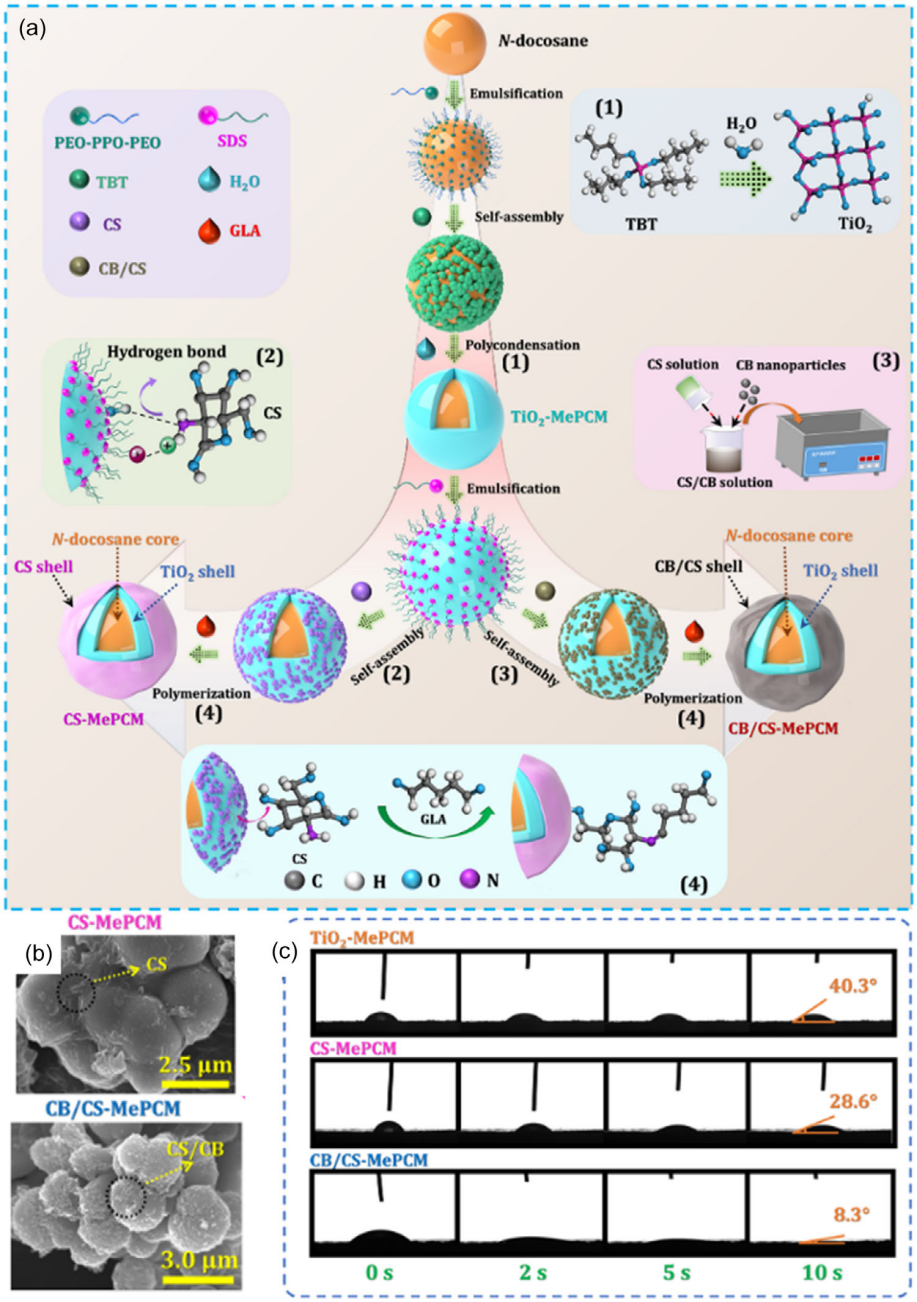
a) Preparation method for multifunctional microcapsules involving TiO_2_ encapsulation, followed by decoration with carbon black (CB) providing photothermal properties and chitosan (CS) providing wettability and antibacterial activity. b) SEM micrographs of the microcapsule samples. c) Wettability of the material evidenced by optical images of water contact angle, as a function of time. Reprinted with permission.^[^ ^235^ ^]^ Copyright 2023, American Chemical Society.

CS is compatible with various encapsulation methods, including spray drying,^[^ ^157^ ^]^ coacervation,^[^ ^158^ ^]^ and electrospinning,^[^ ^159^ ^]^ enabling the formation of both micro‐ and nano‐sized capsules that can be tailored to specific PCM applications.^[^ ^160^ ^]^ Nanoencapsulation with CS is especially useful in biomedical applications. CS's adaptability across encapsulation techniques enhances its application potential, making it a versatile material for PCM systems. Although CS has lower thermal conductivity than certain synthetic materials, it can be effectively combined with thermal conductivity‐enhancing fillers, such as graphene, carbon nanotubes,^[^ ^161^ ^]^ or bioderived carbon materials (as highlighted in this article), metal oxides,^[^ ^156^, ^162^ ^]^ or nitrides, to meet the thermal requirements of high‐performance applications. In summary, CS's renewable origin, film‐forming capacity, antimicrobial effects, and broad compatibility with encapsulation methods make it a viable sustainable alternative for PCM encapsulation applications (**Figure** **5**).

Gelatin is another biopolymer widely used for the encapsulation of PCMs due to its renewable nature, biocompatibility, and versatility in creating stable and effective encapsulation systems^[^ ^163^ ^]^ and advanced materials^[^ ^164^ ^]^ Derived from collagen, typically obtained from animal sources, gelatin is highly regarded for its ability to form strong, flexible films and stable gel structures that make it ideal for encapsulating PCMs in applications where controlled release, environmental safety, and biocompatibility are essential. Similarly to the case of CS, gelatin's film‐forming properties are particularly advantageous in PCM encapsulation as they provide a robust barrier around the PCM core, preventing leakage during phase transitions. Additionally, gelatin exhibits high compatibility with PCMs, ensuring a stable and efficient energy storage system that maintains structural integrity and functionality over multiple thermal cycles.^[^ ^165^ ^]^ As a natural polymer, gelatin decomposes naturally without releasing harmful byproducts,^[^ ^166^ ^]^ making it a preferred choice for environmentally conscious applications.

Furthermore, gelatin is widely compatible with encapsulation techniques,^[^ ^167^ ^]^ such as coacervation, emulsification, and electrospinning, allowing for the production of micro‐ and nano‐sized capsules suitable for a range of PCM applications. Nevertheless, gelatin as a biopolymer for PCM encapsulation has some key disadvantages limiting its application versatility. Firstly, it is highly sensitive to moisture due to its hydrophilic nature, which can cause the shell to swell or degrade in humid environments, leading to PCM leakage. This moisture sensitivity makes gelatin less suitable for outdoor applications or areas with variable humidity. Secondly, gelatin has limited thermal stability and may degrade at higher temperatures, which restricts its use to low‐temperature PCM applications where thermal resilience is not critical. Finally, gelatin's mechanical properties can be problematic as it tends to become brittle, especially under low humidity or with repeated use. This brittleness affects the durability of the encapsulation, leading to potential fractures and loss of PCM containment over time. Most of these disadvantages can be compensated for by the development of various gelatin‐based composites with carbon nanotubes,^[^ ^168^ ^]^ graphite,^[^ ^169^ ^]^ MXenes,^[^ ^170^ ^]^ and other biopolymers.^[^ ^171^ ^]^

**Figure 6:**
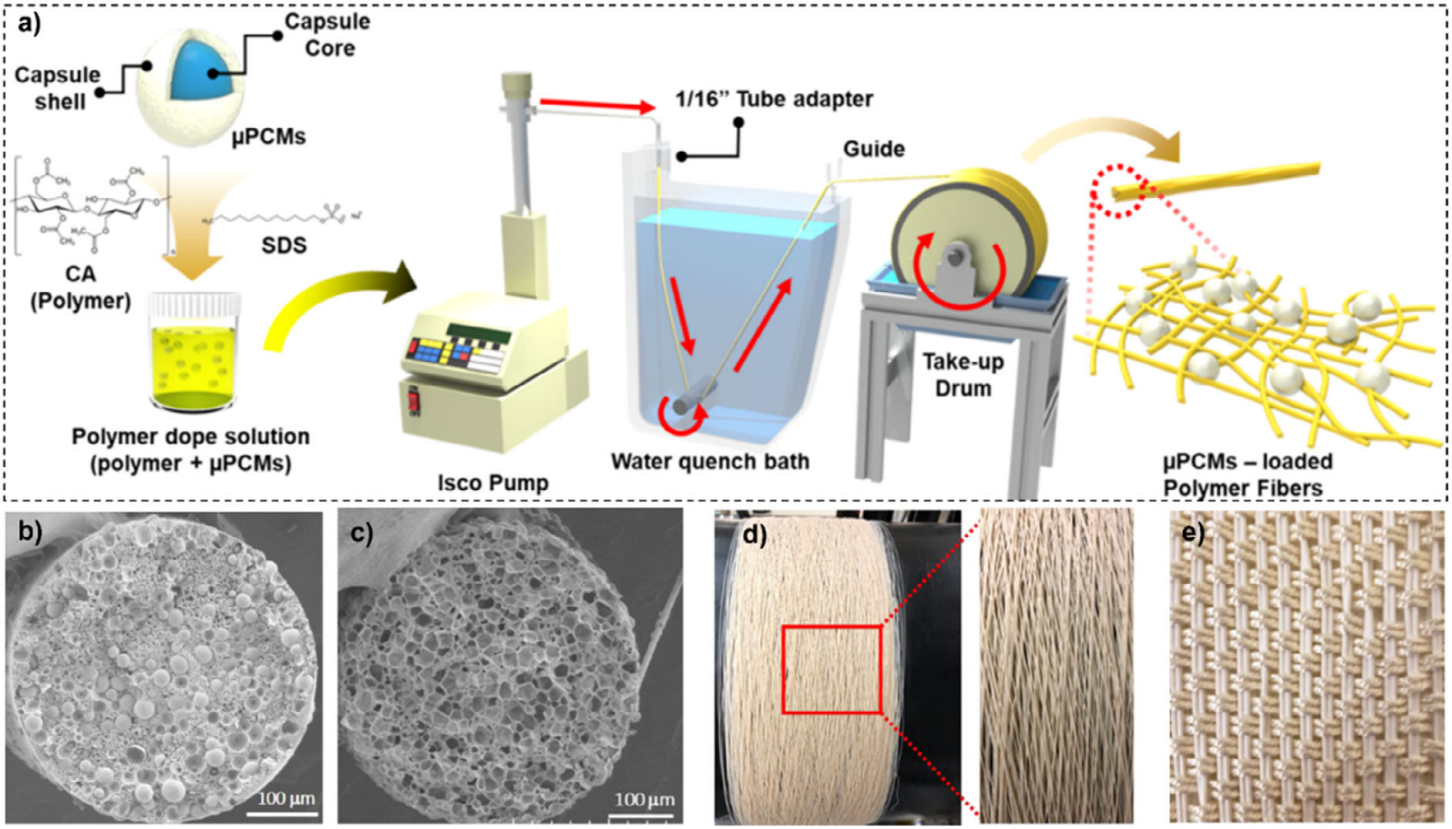
Scheme for a) microencapsulated PCM incorporation into cellulose acetate fibers via the dry‐jet wet‐quench spinning technique. SEM images of b) μPCM‐cellulose acetate fibers and c) μPCM‐cellulose fibers spun with SDS. d) Photographs of pseudo‐non‐woven fabrics made from μPCM‐cellulose fibers. e) Photograph of the woven fabric made from μPCM‐cellulose fibers. Reprinted with permission.^[^ ^236^ ^]^ Copyright 2021, American Chemical Society.

Cellulose is another widely studied biopolymer for PCM encapsulation due to its abundance, renewable origin, and excellent mechanical properties.^[^ ^172^, ^173^ ^]^ Cellulose offers strong mechanical stability, which is crucial for maintaining the integrity of encapsulation shells during repeated phase transitions.^[^ ^174^ ^]^ Its rigidity and durability make it suitable for PCMs used in applications where structural strength is essential. A key advantage of cellulose is its chemical versatility, which allows for surface modifications or chemical functionalization to improve compatibility with various PCMs and encapsulation methods.^[^ ^173^ ^]^ This adaptability enables the development of tailored solutions for specific TES requirements. One of the critical advantages of cellulose is that it can be easily used in additive manufacturing (**Figure** **6**).^[^ ^175^ ^]^

Alginate is a very promising polymer for PCM due to its excellent gel‐forming properties.^[^ ^176^ ^]^ Alginate's versatility lies in its capacity to form microcapsules or beads through simple processes such as ionic gelation, thanks to its ease of processing and adaptability.

**Figure 7:**
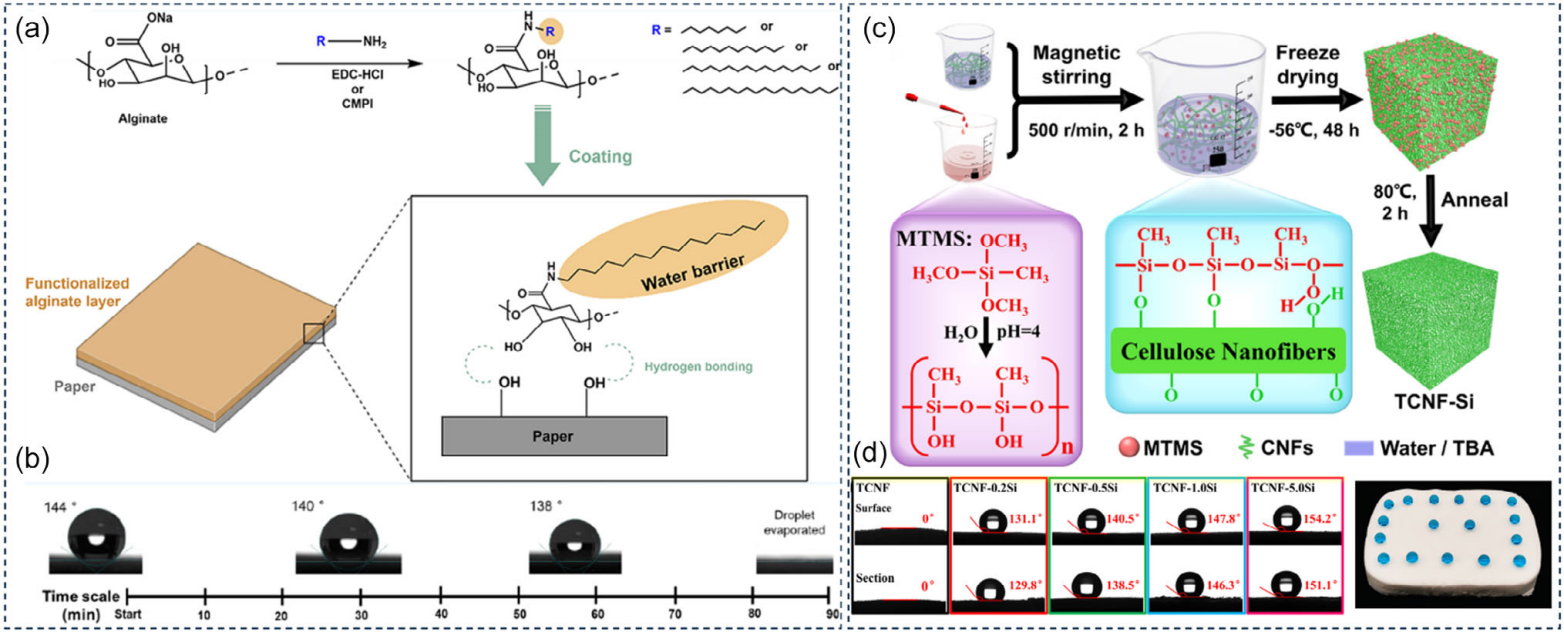
Strategies for increasing the hydrophobility of the biopolymer. a) Scheme for amidation of alginate with alkyl amine and interaction between functionalized alginate and the paper surface to increase its water resistance. b) Water resistance of the material evidenced by optical images of water contact angle, as a function of time (the material remains hydrophobic until the droplet evaporates). Reproduced with permission.^[^ ^181^ ^]^ Copyright, American Chemical Society. c) Scheme for preparation of hydrophobic cellulose nanofibers. d) Water contact angles of the unmodified cellulose nanofibers and cellulose nanofibers with different silylation levels. Reprinted with permission.^[^ ^182^ ^]^ Copyright 2021, American Chemical Society.

All these biopolymers share similar limitations, including moisture sensitivity, low thermal conductivity, and moderate mechanical strength. However, proper design of composites with functional additives can significantly mitigate these challenges and enhance their performance in PCM encapsulation. Functional additives such as carbon materials can improve thermal conductivity, ensuring efficient heat transfer during phase transitions. Reinforcement materials such as silica^[^ ^161^, ^177^, ^178^ ^]^ or clay^[^ ^179^ ^]^ can enhance mechanical strength and durability, increasing the reliability of the encapsulated system. Moisture sensitivity can be addressed by surface modifications^[^ ^180^, ^181^, ^182^, ^183^, ^184^ ^]^ (**Figure** **7**) or blending biopolymers with hydrophobic materials^[^ ^185^ ^]^ to create moisture‐resistant shells. Additionally, incorporating crosslinking agents or copolymers can improve the structural stability and reduce degradation in humid conditions. By carefully tailoring the composition and processing techniques, biopolymer‐based PCM encapsulation systems can achieve the desired balance of sustainability, functionality, and durability, making them suitable for a wide range of advanced TES applications (**Table** **6**).

**Table 6:** Polymer selection for PCM encapsulation.

Desired requirement	Recommended polymer types	Encapsulation method	Suitable PCM types	Applications
Biodegradability and low toxicity	Alginate, CS, gelatin	Spray drying, coacervation	Organic and inorganic PCMs	Food storage,^[^ ^176^ ^]^ textiles,^[^ ^257^ ^]^ agriculture^[^ ^258^ ^]^
High thermal conductivity	PLA, CS with fillers (carbon nanotubes, graphene, graphite, boron nitride)	Electrospinning, spray drying		Electronics, waste heat recovery, renewable energy storage, cooling systems^[^ ^235^, ^258^ ^]^
High mechanical stability	PLA, starch blends, CS blends with PVA^[^ ^161^ ^]^and fillers (silica, clay, CB, etc.)	Interfacial polymerization,^[^ ^259^ ^]^ coacervation,^[^ ^260^ ^]^		Construction, automotive
Cost‐effectiveness	Starch, alginate	Spray drying^[^ ^261^, ^262^ ^]^		Large‐scale, low‐cost energy storage

## 5 How to Fabricate a Sustainable PCM?

**Figure 8:**
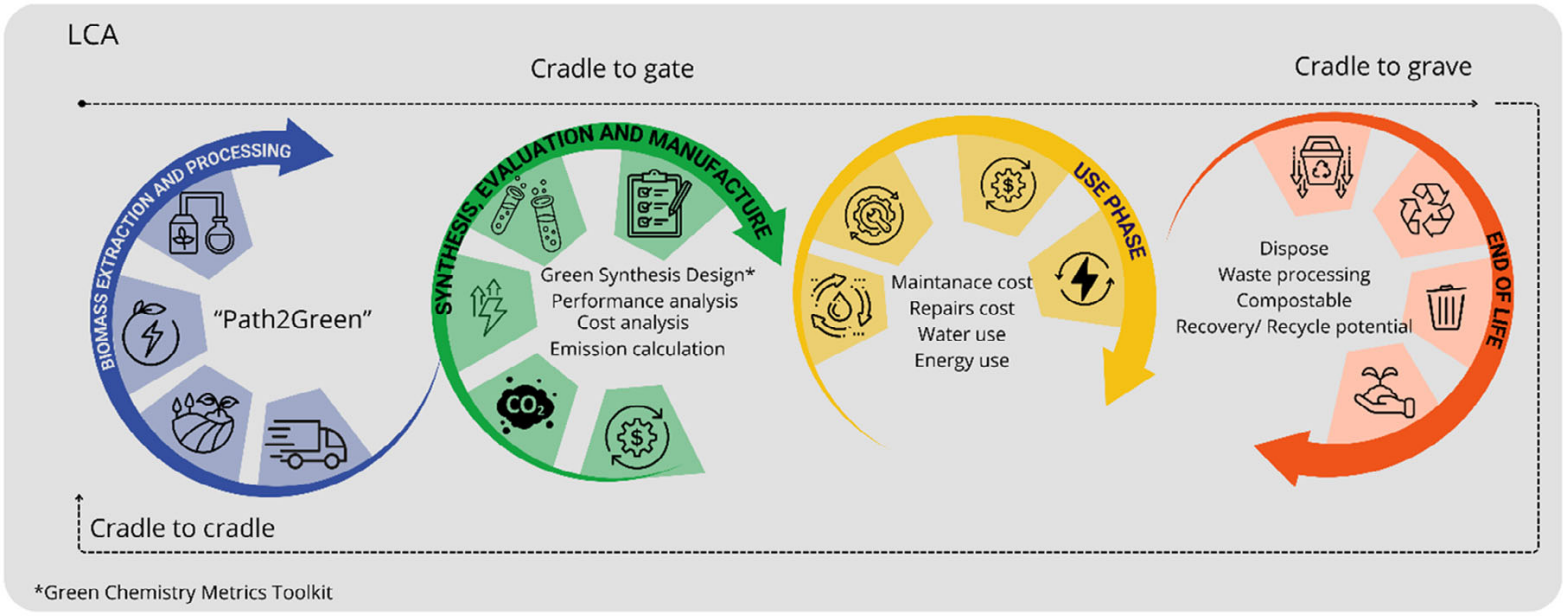
Four stages of the LCA process, highlighting tools and analyses for evaluating the sustainability of PCMs.^[^ ^194^, ^195^ ^]^

The sustainability of PCMs is not solely determined by the source of the precursors. Aligned with the principles of a circular economy, the entire material value chain, including unit operations, must be considered, from design and fabrication, through operation, to recycling, recovery, and disposal. Various tools are available to assess material sustainability, which can also be applied to PCMs. Among these, the most comprehensive method is life cycle assessment (LCA), which analyzes the environmental impact of the material during its entire life (**Figure** **8**).^[^ ^186^ ^]^

The LCA comparison between biomass‐derived PCMs and conventional PCMs reveals a complex picture with varying outcomes depending on specific materials, processing methods, and system boundaries. Based on recent work^[^ ^68^, ^187^, ^188^, ^189^ ^]^ we can highlight several key points below.

### 5.1 Environmental Impact Comparison

Biomass‐derived PCMs do not universally outperform conventional PCMs in environmental impact assessments. Fabiani et al.^[^ ^190^ ^]^ found that raw palm oil‐based PCM produced significantly higher emissions (2.772 kgCO_2_‐eq/kg) compared to paraffin wax (0.207 kgCO_2_‐eq/kg) due to the environmental burden of harvesting and oil extraction processes. However, when waste palm oil from the food industry was assessed instead, emissions were dramatically reduced to 0.053 kgCO_2_‐eq/kg, highlighting the potential advantages of utilizing recycled bio‐based materials.

Similarly, Baylis and Cruickshank^[^ ^189^ ^]^ compared coconut oil to paraffin PCMs and concluded that although coconut oil exhibited lower embodied carbon, paraffins demonstrated greater thermal storage capacity, resulting in greater annual emissions reductions and consequently lower lifecycle emissions overall in three North American locations. These findings underscore the critical importance of case‐by‐case comparisons when evaluating the environmental performance of PCMs, as factors including raw material sourcing, manufacturing processes, and application‐specific thermal properties significantly influence lifecycle impacts.

The potential environmental impact generated during the production or utilization process can be remediated by the cyclability of the material during its operation. Recently, Piper et al. estimated CO_2_ emissions associated with using an isocyanate‐based PCM in relation to lifetime energy stored and delivered, assuming a conservative 5‐year lifespan.^[^ ^82^ ^]^ The CO_2_ emissions from using this PCM were estimated to be 0.012 kg kW^−1^ h^−1^, being substantially lower compared to the emissions from coal‐fired power per kW h (≈1 kg kW^−1^ h^−1^) and lithium‐ion battery use (≈0.029 kg kW^−1^ h^−1^), assuming a battery lifetime of 2500 cycles. This holistic approach, which evaluates not only the environmental impact but also the “rate of return” by considering the performance of the PCM in specific applications, can help to determine if its use is justified or not.

### 5.2 Energy Consumption

The energy consumed during the production of biomass‐derived PCMs remains a significant concern. As noted by Yani et al.^[^ ^191^ ^]^ ≈41% of emissions is associated with coconut oil production stem from energy‐intensive processing in crude coconut oil plant systems. The embodied energy of bio‐based PCMs could be significantly reduced through 1) electrification and efficiency improvements in extraction processes, 2) use of non‐fossil fuel boilers, 3) cleaner electricity sources, and 4) optimized energy usage throughout production.

### 5.3 Waste Generation and Circular Economy Benefits

A major advantage of biomass‐derived PCMs is their potential integration into circular economy models. Several studies highlight opportunities for utilizing waste streams. Secondary‐use bio‐PCMs from food industry waste can achieve substantially lower emissions than primary‐use bio‐PCMs or paraffins.^[^ ^190^ ^]^ Bio‐based PCMs may offer improved end‐of‐life options through biodegradability or recyclability, although this aspect requires more detailed investigation as biodegradability cannot be assumed simply due to biomass origin.^[^ ^75^ ^]^

### 5.4 Advantages of Biomass‐Derived PCMs

Bio‐based PCMs offer compelling advantages over conventional alternatives despite mixed LCA results. These materials exhibit significantly lower flammability than paraffin‐based PCMs, maintaining fire resistance while improving thermal mass in building applications.^[^ ^192^ ^]^ From palm kernel oil, dodecanoic acid demonstrates rapid carbon payback in solar thermal systems, while algae‐derived ethyl hexadecanoate shows substantial carbon sequestration potential.^[^ ^193^ ^]^ With heat storage capacities comparable to traditional PCMs (180–187 J g^−1^) or even exceeding them if sugars or sugar alcohols are considered (300 J g^−1^) and freedom from phase separation issues that plague inorganic alternatives, bio‐based PCMs provide reliable thermal performance across diverse temperature ranges, from room‐temperature buffering to solar hot water systems. Though production energy requirements remain high for some bio‐based PCMs, particularly algae‐derived varieties, their environmental profile continues to improve with advancing technology, making these renewable materials increasingly viable alternatives for TES applications.

While detailed LCA is often beyond the scope of articles focusing on the design of new PCMs and their physicochemical properties, the development of PCMs, which is essential to the results of any LCA, can be directed by the Principles of Green Chemistry (Figure 8).

Isolating PCMs (type 0) or precursors for PCMs (types 1, 2, and 3) from biomass involves an extraction step. Although extraction processes have been used since ancient times and are generally considered nonharmful, the recent surge in interest in bioderived products has significantly intensified these practices. This highlights the urgent need to develop sustainable methods that prevent the depletion of primary resources, support biodiversity, and minimize waste generation and energy consumption.^[^ ^194^ ^]^ Recently, M. de Souza Mesquita et al. formulated 12 principles of green extraction processes (Path2Green), providing metrics that offer easy evaluation of the environmental impact of specific extraction activities as well as unit operation selection in the most sustainable and cost effective way.^[^ ^194^ ^]^ Moreover, the preparation of PCMs that involve chemical modifications of the biomass feedstock (types 1 and 2) can be guided by the Principles of Green Chemistry summarized by J. Clark's Green Chemistry Metrics Toolkit.^[^ ^195^ ^]^ This tool analyzes the reagents, catalysts, and solvents used regarding quantity and quality (defined through their health and safety parameters), key reaction conditions (reaction temperature, pressure, and time), and finally the purification method to provide recommendations for the development of sustainable procedures. An example of such green chemistry principles dictating synthesis is the development of tartaric acid‐derived PCMs, recently reported by Gwóźdź et al.^[^ ^75^ ^]^ PCMs melting between 67 and 94 °C, with high Δ*H*_f_ values reaching 221 J g^−1^, were synthesized through esterification reactions conducted solventless at 70 °C, with a heterogeneous, reusable catalyst, followed by purification through crystallization from ethanol, all of which could be assigned a green flag according to the toolkit.^[^ ^195^ ^]^ Moreover, some principles included in this toolkit can guide the fabrication of the PCM composite, that is, the encapsulation step, including reagents and solvent selection, as well as the preferred reaction conditions.

Furthermore, to meet the criteria for sustainable materials, such materials should be reusable or recyclable at the end of their initial lifecycle.^[^ ^31^ ^]^ For PCMs, this specifically relates to their ability to repeatedly store and release energy over multiple cycles. Notably, when the performance of a PCM diminishes over successive cycles, recrystallization of the material may help in restoring its original functionality. When it comes to composites, a strategy to extend their lifecycle includes grinding, PCM separation, and recrystallization, which collectively enable both the regaining of performance characteristics and the recovery of other components, contributing to the circular economy with a cradle‐to‐cradle design. When considering the disposal of materials, they should meet environmental standards by demonstrating high biodegradability and low toxicity. Insights into these parameters can help assess the potential environmental risks associated with new materials. To the best of our knowledge, no comprehensive data on these parameters have been compiled specifically for PCMs. However, many organic compounds used as PCMs have been studied for their environmental impact, including their toxicity toward specific organisms and their potential for complete mineralization into CO_2_, H_2_O, and microbial biomass.^[^ ^75^, ^196^, ^197^ ^]^ Finally, techno‐economic analysis or Life Cycle Cost Analysis (LCCA) can be valuable tools for assessing the profitability of using a specific PCM in a given application. For example, Jacob et al. evaluated the capital investment cost recovery period for photovoltaic thermal systems integrated with PCM. These researchers estimated the payback period for systems based on a mixture of capric acid and lauric acid or animal fat, ranging from 3 to 4 years.^[^ ^198^ ^]^ Another study by Lee et al. presented a techno‐economic evaluation of a salt hydrate‐based PCM in a thermal battery, demonstrating that integrating such a battery is an effective strategy for improving the efficiency of cooling systems, particularly during periods of high cooling demand.^[^ ^199^ ^]^

### 5.5 Economic Feasibility of Biomass‐Derived PCMs

The economic feasibility of biomass‐derived PCMs depends on numerous interconnected factors spanning production, application, and long‐term performance. Biomass‐derived PCMs benefit from abundant and renewable feedstock sources, positioning them favorably in the expanding bioeconomy. According to Mousavi‐Avval et al.^[^ ^200^ ^]^ the global bioeconomy market size excluding food and feed was valued at $3.43 trillion in 2018 and is projected to increase to $5.05 trillion by 2030, indicating the growing economic viability of bio‐based products generally. Biomass feedstock supply remains competitive, with agricultural and forest sectors providing over 82% of current biomass, while the construction sector, a primary application area for PCMs, is projected to experience the largest growth in biomass use at a compound annual growth rate of 8.8% from 2018 to 2030.

Feedstock selection significantly impacts the economic viability of biomass‐derived PCMs. Using waste or by‐product biomass, such as sunflower and pomelo peel‐derived porous carbons as demonstrated in recent studies, can significantly reduce raw material costs compared to synthetic PCMs. This approach also aligns with circular economy principles, creating value from materials that would otherwise be discarded.

Processing costs represent another important economic consideration. PCM processing methods including shape stabilization and thermal conductivity enhancement add to manufacturing expenses, but they also improve performance and can reduce overall system costs during the operational phase. The processing techniques must be optimized to achieve the best cost‐performance ratio for specific applications.

Scale‐up considerations present both challenges and opportunities. As described earlier, most PCM leakage measurements in current research were taken for periods shorter than 2 h, with few studies reporting longer‐term stability over 100–200 thermal cycles. This indicates a need for more extensive testing and potentially higher initial validation costs before large‐scale commercial implementation can be realized. Robust long‐term performance data will be crucial for economic feasibility assessments and market acceptance.

The economic benefits of biomass‐derived PCMs extend beyond direct material costs. High porosity with open pore networks allows high PCM loading and retention over time, potentially providing greater energy storage per unit cost compared to traditional PCMs. Bio‐based products generally help reduce petroleum consumption and greenhouse gas emissions, providing potential carbon credit benefits or meeting increasingly stringent building regulations. For end users, efficient TES systems using biomass‐derived PCMs could significantly reduce heating and cooling energy requirements in buildings, offering substantial operational cost savings over time.

As petroleum prices fluctuate and environmental regulations tighten, the relative cost‐competitiveness of biomass‐derived PCMs is likely to improve compared to petroleum‐based alternatives. This trend may accelerate adoption in cost‐sensitive construction and manufacturing sectors.

A fundamental economic trade‐off exists between PCM loading and thermal conductivity. As noted in our analysis, larger PCM loading tends to reduce the thermal conductivity of the shape‐stabilized PCM. This technical challenge requires finding an economic optimum between these competing factors. Controlling the structure, texture, and porosity of the carbon support offers a pathway to achieve a good compromise between properties, which would have direct economic implications for manufacturing processes and end performance.

In conclusion, while specific cost figures for biomass‐derived PCMs vary widely based on feedstock, processing methods, and scale, the growing bioeconomy market and advances in biomass utilization suggest improving economic feasibility. The key to commercial viability will be optimizing the balance between material performance (thermal conductivity, stability, and energy storage capacity) and production costs, while leveraging the sustainability benefits that biomass‐derived PCMs offer compared to traditional PCMs. Further research on large‐scale production methods and long‐term performance will be essential to fully realize the economic potential of these promising materials.

## 6 Applications

**Figure 9:**
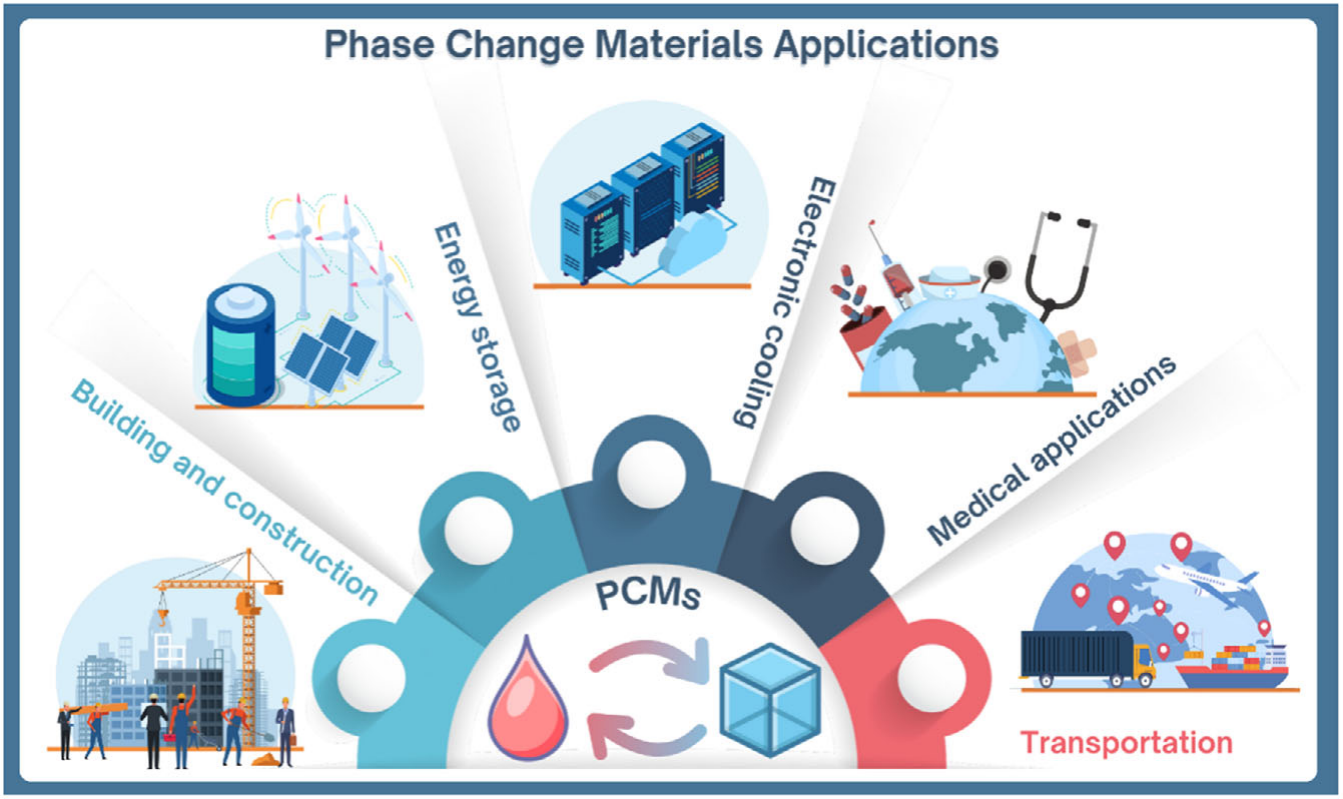
Possible application areas for bio‐PCMs across various industries.

PCMs have a broad range of applications from building and construction, through textiles, to cold‐chain transportation (**Figure** **9**).^[^ ^201^, ^202^ ^]^ Their unique ability to absorb, store, and release large amounts of thermal energy at nearly constant temperatures makes them ideal for TES and temperature regulation applications. As global energy demands rise and the need for sustainable solutions becomes more pressing, PCMs offer promising pathways for energy efficiency and thermal management. The selection of appropriate PCMs for specific applications depends critically on their thermophysical properties, with melting temperature being the most crucial parameter that must align with the intended operating conditions.

### 6.1 Building and Construction

PCM integration in building and construction represents one of the most significant advances in sustainable architecture and construction.^[^ ^203^, ^204^ ^]^ When integrated into modern buildings, PCMs can be microencapsulated and incorporated across multiple building elements, from windows^[^ ^205^ ^]^ and floors,^[^ ^206^, ^207^ ^]^ to fundamental construction materials, such as concrete, gypsum, and plaster.^[^ ^208^, ^209^ ^]^ This type of applications constitute passive building application where materials work through a natural thermal regulation cycle: during peak daytime temperatures, they absorb excess heat through melting and then release this stored energy at night as temperatures drop, and the material resolidifies. This passive temperature control mechanism significantly reduces reliance on conventional heating, ventilation, and air‐conditioning systems, enhancing building energy efficiency and sustainability.^[^ ^210^ ^]^ On the other hand, active PCM systems enhance thermal control through the integration of auxiliary equipment, such as fans and pumps, which regulate heat transfer by controlling the movement of heat transfer media. This approach finds widespread application in building services, including space heating and cooling systems, air conditioning, and domestic hot water supply. The active control allows for more precise temperature regulation and optimized energy utilization compared to passive systems.

### 6.2 Energy Storage

Currently, society is standing at the threshold of a significant energy transformation, poised to turn away from fossil fuels and pivot towards renewable energy resources to create a more sustainable future. This global shift is driven by the urgent need to mitigate climate change and reduce carbon emissions.^[^ ^3^ ^]^

TES, which can capture and retain heat from solar,^[^ ^211^ ^]^ industrial processes, waste heat recovery,^[^ ^212^ ^]^or excess electricity generation, offers unique advantages in terms of low cost, efficiency, scalability, and potential for integration with existing energy systems, being very attractive for surplus renewable energy.^[^ ^14^, ^213^ ^]^

An innovative approach to renewable energy storage comes in the form of Carnot battery technology, which represents a cost‐effective and location‐flexible energy storage solution. This technology ingeniously converts electrical energy into thermal energy, storing it in readily available, low‐cost materials for later utilization. A Carnot battery operates through a two‐phase cycle. During the charging phase, renewable energy powers a heat pump that transfers thermal energy from a cold reservoir to a hot reservoir, where it can be stored in TES materials. The discharge phase reverses this process: the stored thermal energy drives the heat pump in reverse, functioning as an organic Rankine engine to generate electricity. This process also produces valuable lower‐temperature heat (80–120 °C) that can be harnessed for various practical applications, such as district heating or industrial processes. This technology has already demonstrated impressive performance metrics, achieving power‐to‐power round‐trip efficiencies of 72%.^[^ ^214^ ^]^ However, it currently exists only at the theoretical level. As research and development continue to optimize this relatively new process, projections suggest that efficiencies approaching 100% may be attainable. This remarkable potential, combined with the system's simplicity and use of abundant materials, makes Carnot batteries a particularly promising solution for grid‐scale energy storage.

A key advantage of this system lies in its dual‐output nature: not only does it provide electrical power when needed, but it also produces useful thermal energy that can be integrated into existing heating systems, thereby increasing the overall system efficiency through cogeneration.

### 6.3 Textiles and Personal Comfort

The textile industry embraced PCMs to create smart fabrics and clothing that actively regulate body temperature.^[^ ^147^, ^215^, ^216^ ^]^ These materials are typically incorporated through microencapsulation into fiber structures or fabric coatings or sold as separate heating/cooling packs.^[^ ^217^, ^218^ ^]^ When body temperature rises, the PCMs absorb excess heat and melt, providing a cooling effect. Conversely, as body temperature drops, the PCMs solidify and release stored heat, maintaining comfort.

This technology has found particular success in outdoor and athletic wear^[^ ^219^ ^]^ and space suits^[^ ^217^, ^220^ ^]^ where temperature regulation is crucial for performance and comfort. PCM‐enhanced textiles are also used in bedding materials,^[^ ^221^ ^]^ where they help maintain optimal sleeping temperatures throughout the night.

### 6.4 Electronic Cooling

The challenge of managing heat in electronic devices has grown with increasing processing power and miniaturization. PCMs offer an elegant solution by absorbing heat during periods of high computational load and releasing it when the device is less active. This helps prevent thermal throttling and extends the device's lifespan by avoiding temperature spikes.^[^ ^222^, ^223^, ^224^ ^]^

In data centers, PCM‐based cooling systems help maintain optimal operating temperatures while reducing the energy consumption of traditional air conditioning systems. The technology is particularly effective in managing periodic heat loads and providing backup cooling during power outages.^[^ ^225^ ^]^

### 6.5 Transportation

PCMs are used in the transportation industry to solve complex temperature control challenges, particularly in the shipping of temperature‐sensitive goods.^[^ ^226^ ^]^ In refrigerated transport, PCM‐based systems provide a reliable backup to mechanical cooling systems and can maintain stable temperatures even during power outages. This is particularly crucial for food transport, where temperature fluctuations can lead to spoilage and significant economic losses.

In the pharmaceutical cold chain, PCMs have become indispensable for maintaining the precise temperature ranges required for biological products and vaccines.^[^ ^226^, ^227^ ^]^ These materials can be engineered to maintain specific temperature ranges, from deep freezing to room temperature, ensuring product efficacy throughout the distribution process. The reliability of PCM‐based shipping containers has made them the preferred choice for transporting valuable biological materials and temperature‐sensitive medications.

PCMs are also studied and used in the application of spacecraft avionics.

### 6.6 Medical Applications

In healthcare, PCMs are used in the storage of biological materials.^[^ ^228^, ^229^ ^]^ Blood products, vaccines, and tissue samples require precise temperature control, and PCM‐based storage solutions provide this with greater reliability than traditional methods.^[^ ^230^ ^]^ These systems are particularly valuable in regions with unreliable power supplies or during emergency situations where maintaining the cold chain is challenging.

PCMs are also widely used in therapeutic applications.^[^ ^231^ ^]^ Hot and cold therapy packs using PCMs can maintain therapeutic temperatures for longer periods than traditional solutions. These products are particularly valuable in physical therapy and rehabilitation settings, where consistent temperature application is crucial for treatment effectiveness. Other applications include drug delivery,^[^ ^232^ ^]^ osteoporosis treatment,^[^ ^233^ ^]^ and tumor thermochemotherapy.^[^ ^234^ ^]^

The versatility of PCMs continues to drive innovation across these sectors, with new applications emerging as the technology advances. The ability to engineer PCMs with specific melting points and thermal storage capacities makes them adaptable to an ever‐widening range of uses, from everyday consumer products to critical medical and industrial applications.^[^ ^74^ ^]^

## 7 Summary and Outlook

The development of biomass‐derived PCMs represents a crucial advance in sustainable materials science, offering promising solutions for TES, while addressing pressing environmental challenges.

With this perspective article, we aimed to provide a guideline for material selection in line with green chemistry principles and circularity criteria. Nevertheless, it is worth emphasizing that the study of materials that may not necessarily be environmentally ideal remains valuable. These materials offer critical insights into understanding structure‐property relationships, indispensable for developing more effective and sustainable PCMs in the future.

Although biobased materials have not yet reached the same level of large‐scale commercialization as petroleum‐based materials, their availability continues to improve with technological advancements in biomass processing methods, including extraction, purification, and chemical modifications. This review has demonstrated that naturally abundant biomass resources can already effectively yield PCMs, PCM supports, and encapsulants with performance characteristics comparable to or exceeding their fossil‐based equivalents. Firstly, the wide variety of biomass feedstocks available provides numerous pathways for developing PCMs from type 0 to type 3 with tailored thermal properties spanning across a temperature range from −30 to 150 °C, enabling a diverse range of applications. Secondly, the integration of biomass‐derived carbons and biopolymers for SSPCMs and as encapsulation materials completes the toolkit for fully bioderived systems that additionally improve some of the inherent properties of biobased PCMs, such as poor thermal conductivity, leakage, supercooling, and the need for protection from oxidation. Bio‐derived systems may be also susceptible to microbial contamination, requiring stabilizers or preservatives.

Nevertheless, PCMs are emerging as promising candidates for advanced applications beyond traditional thermal storage. Their properties, including reversible volume change, response to thermal stimuli, and optical transparency, make them particularly suitable for integration into intelligent, stimuli‐responsive materials for robotics or electronics. The ability to source PCMs and their components from renewable raw materials opens new horizons for more sustainable functional materials for high‐tech applications. However, significant challenges remain to be addressed, which are not only related to biobased PCMs but to all PCMs in general. Future research should focus on 1) improving long‐term stability and cycling performance; 2) performing LCA, which analyzes the environmental impact of the material during its entire life; 3) following the Principles of Green Chemistry while designing materials and their synthetic procedures at the laboratory level; and 4) testing materials in real‐life applications, including their compatibility with storage containers.

## Conflict of Interest

The authors declare no conflict of interest.
